# Cancer metastasis under the magnifying glass of epigenetics and epitranscriptomics

**DOI:** 10.1007/s10555-023-10120-3

**Published:** 2023-06-28

**Authors:** Maxime Janin, Veronica Davalos, Manel Esteller

**Affiliations:** 1 Cancer Epigenetics Group, Josep Carreras Leukaemia Research Institute (IJC), IJC Building, Germans Trias I Pujol, Ctra de Can Ruti, Cami de Les Escoles S/N, 08916 Badalona, Barcelona, Spain; 2 Centro de Investigacion Biomedica en Red Cancer (CIBERONC), Madrid, Spain; 3 Institucio Catalana de Recerca I Estudis Avançats (ICREA), Barcelona, Catalonia Spain; 4 Physiological Sciences Department, School of Medicine and Health Sciences, University of Barcelona (UB), Barcelona, Catalonia Spain

**Keywords:** Epigenetic, Epitranscriptomic, Cancer metastasis, Cancer treatment

## Abstract

Most of the cancer-associated mortality and morbidity can be attributed to metastasis. The role of epigenetic and epitranscriptomic alterations in cancer origin and progression has been extensively demonstrated during the last years. Both regulations share similar mechanisms driven by DNA or RNA modifiers, namely writers, readers, and erasers; enzymes responsible of respectively introducing, recognizing, or removing the epigenetic or epitranscriptomic modifications. Epigenetic regulation is achieved by DNA methylation, histone modifications, non-coding RNAs, chromatin accessibility, and enhancer reprogramming. In parallel, regulation at RNA level, named epitranscriptomic, is driven by a wide diversity of chemical modifications in mostly all RNA molecules. These two-layer regulatory mechanisms are finely controlled in normal tissue, and dysregulations are associated with every hallmark of human cancer. In this review, we provide an overview of the current state of knowledge regarding epigenetic and epitranscriptomic alterations governing tumor metastasis, and compare pathways regulated at DNA or RNA levels to shed light on a possible epi-crosstalk in cancer metastasis. A deeper understanding on these mechanisms could have important clinical implications for the prevention of advanced malignancies and the management of the disseminated diseases. Additionally, as these *epi-alterations* can potentially be reversed by small molecules or inhibitors against *epi-modifiers*, novel therapeutic alternatives could be envisioned.

## Introduction

Cancer is a disease defined by the uncontrolled proliferation of cells that have activated oncogenic programs to avoid endogenous controls. Initially defined by the affected tissue of origin, the development of high-throughput technologies to molecularly characterize tumor samples has accelerated and expanded our understanding on tumorigenesis, revealing a plethora of cancer-specific changes or signatures of potential use as biomarkers to define diagnosis and prognosis and improve treatment decisions [1, 2]. A critical stage in cancer progression is the spread of cancer cells to other parts of the body through the bloodstream or lymphatic system to invade other organs or tissues, termed metastasis. Cancer metastasis is a multistep biological process known as the invasion-metastasis cascade, and it is a marker of cancer progression with poor clinical consequences for patients, as at least two-thirds of cancer-related deaths are caused by metastasis [3, 4]. Although it was considered that metastasis occurs at final steps of tumor progression, recent studies have shown that metastasis can arise early in tumorigenesis [5–7]. When the clones escape early, there is a higher genetic divergence between the primary tumor and metastases. Also, clones can disseminate as single circulating tumor cells (CTC) or in clusters to seed monoclonal and polyclonal metastases, respectively [8].

The epidemiology of metastasis differs among types; for example, the incidence of brain metastases is around 20%, originated mostly from primary breast, lung, and melanoma tumors, and affecting approximately 200,000 patients with cancer each year in the USA [9]. For bone metastases, the incidence is around 5%, mainly derived from primary lung tumors [10]. Regarding lung metastases, the synchronous ones, meaning occurring during the 6 months following the first diagnostic, mainly originate from primary lung cancer, followed by colorectal, kidney, pancreatic, and breast primary tumors. The age-adjusted incidence rate of synchronous lung metastasis was around 18 per 100,000 between 2010 and 2015 [11]. The patient survival also differs depending on primary sites and cancer subtypes. For example, the 5-year survival for patients with liver metastases from colorectal cancer (CRC) is around 63%, but falls at 5–15% for pancreatic cancer [12–14]. Moreover, in cancers of unknown primary (CUP), a term used to describe a heterogeneous group of metastatic tumors for which a standardized diagnostic work-up fails to identify the site of primary origin at the time of diagnostic work-up [15–17], survival is generally worse than in primary cancers with metastasis to the same organ where CUP is detected [12]. Regarding cancer subtypes, the median survival from time of first distant metastasis is 26 months for luminal-A breast cancer subtype compared to only 6 months for basal-like subtype [18].

Mechanistically, during cancer progression, external negative selective pressures for instance from tumor microenvironment or cancer treatment shape intratumor heterogeneity. This heterogeneity is evidenced by the acquisition of not only additional genetic mutations, but also epigenetic and epitranscriptomic changes, which can now be dissected at unprecedented resolution using novel single-cell technologies. These mechanisms promote the expression of oncogenic programs that can drive aggressiveness through phenotypic changes that improve the cellular ability to disseminate and invade distant organs. Strikingly, although many genetic mutations have been linked to tumor initiation and progression, no specific driver gene mutations exclusive to metastases have been identified to date [8]. A more related factor is chromosomal instability, with up to 80% of whole genome doubling in metastases for some tumor types; nevertheless, chromosomal instability can also be found in around 30% of primary tumors [8]. The lack of a direct genetic connection to metastasis suggests that other mechanisms may play a more significant role in driving the decision of cells to engage in this process. Moreover, the pattern of affected organs is remarkably variable depending on the cancer type, indicating that intrinsic properties of the tumor and the composition of host-organ microenvironment are important determinants of the sites of metastasis, as well as the organ-specific circulation pattern [19, 20]. This observation implies that essential mechanisms control the interactions between cancer cells and specific site microenvironment for induction of metastatic competence. The induction of the aforementioned oncogenic program leading to the metastatic cascade is usually driven by the repression of metastasis suppressor genes. For example, the metastasis suppressor genes *CREB3L1* (cAMP-responsive element binding protein 3 like 1) and *MTSS1* (metastasis suppressor 1) were recently found repressed in triple-negative breast cancer (TNBC) [21]. Also in breast cancer, the metastasis suppressor gene *SCN4B*, encoding for the sodium channel β4 subunit, has a reduced expression comparing with normal tissue, leading to increased RhoA activity, cell migration and invasiveness, and metastatic spreading [22]. In order to rewire gene expression on an adaptive and selective advantage manner, the plasticity and reversibility of epigenetic and epitranscriptomic mechanisms make them ideal orchestrators of the dynamic process of metastasis [23].

Along this review, we first briefly introduce the main mechanisms driving metastasis, and next summarize relevant *epi-regulations* described to date on this process, highlighting parallel epigenetic and epitranscriptomic alterations implicated on the same pathways for specific cancer metastases. Finally, we briefly comment therapeutic opportunities, as small molecules against key *epi-modifiers* have been developed and could have the potential to disrupt the metastatic process.

### The metastatic cascade

**Fig. 1:**
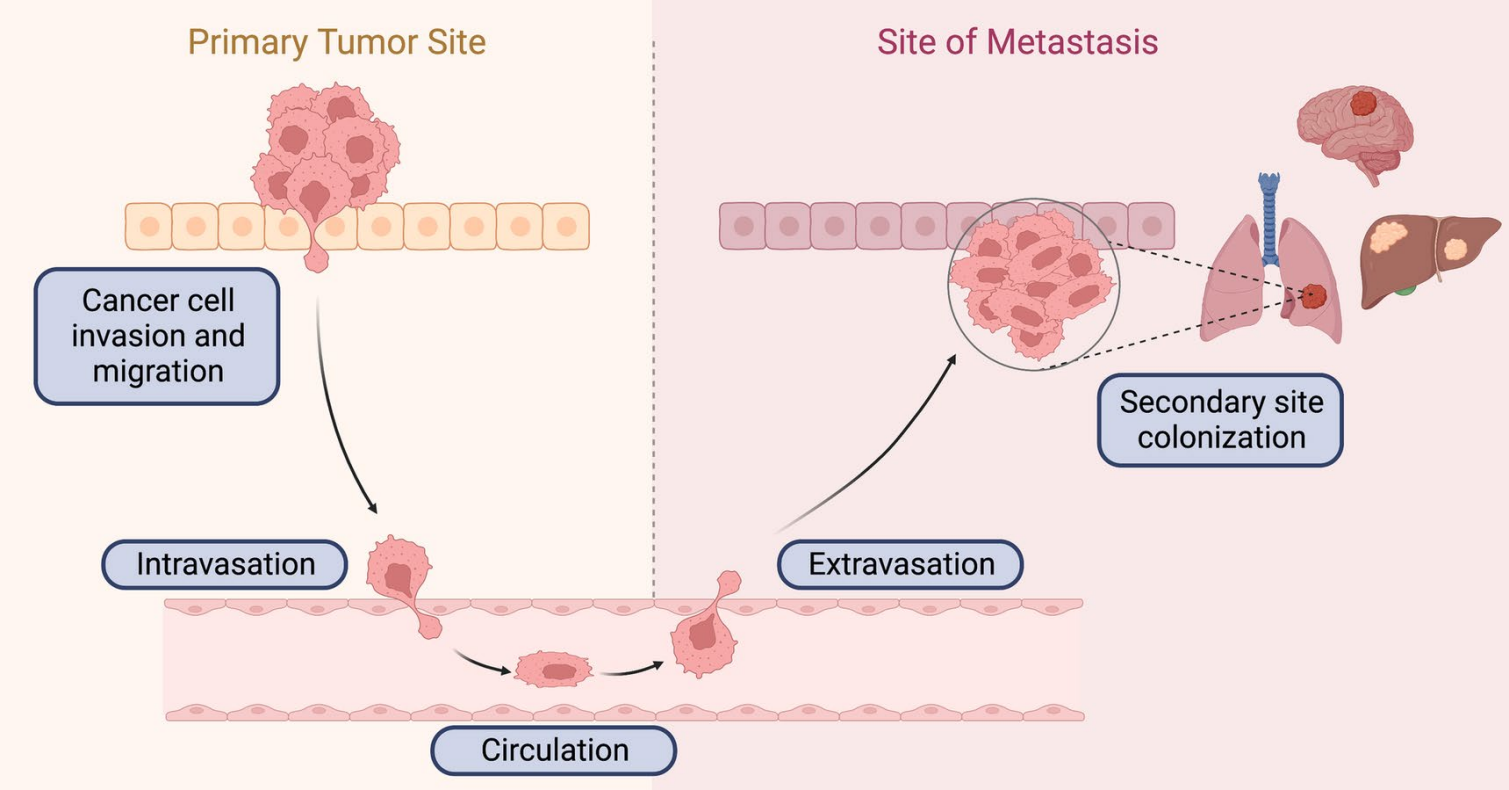
Schematic overview of the metastatic cascade. Simplified view highlighting the main steps of the metastatic cascade, a complex process that involves the inhibition of metastasis suppressor genes and the activation prometastatic/oncogenic signaling pathways that control survival, proliferation, angiogenesis, and invasion. Created with BioRender.com

The metastatic cascade is defined by five different steps that allow tumor cells to leave the tissue of origin, spread to a distant area of the body, and finally colonize and grow in a different tissue (Fig. 1) [24, 25]. This process is extremely inefficient and can be aborted at several points. Indeed, the vast majority of cancer cells never leave the primary tumor, and of those that manage to enter into the circulation, most fail to colonize distant organs or are recognized by immune cells and abolished [26, 27]. Therapies that decrease the survival of metastatic cells in the vascular system have been shown to lower the risk of developing metastasis [28], but not all the steps of the metastatic cascade are amenable to therapeutic intervention, underlying the importance of identifying the drivers of the initial steps to develop effective interventions against the metastatic process [29].

Mechanistically, the first step, joining invasion and migration, consists of the separation of the tumor cells from their neighborhood cells in the primary tumor. One of the key mechanisms involved in the first steps of metastasis is the epithelial to mesenchymal transition (EMT), a biologic process that enables polarized epithelial cells to acquire a mesenchymal phenotype with enhanced migratory capacity, invasiveness, elevated resistance to apoptosis, and increased production of extracellular matrix components [30]. This process is mediated by transcription factors belonging to three distinct families: the Snail family (SNAIL1/SNAIL, SNAIL2/SLUG, and SNAIL3/SMUC), the Zeb family (ZEB1, ZEB2), and the b-HLH family (TWIST1, TWIST2) [31], as well as other factors that enhance cancer cell invasion through the crosstalk with the tumor microenvironment [32, 33]. These EMT transcription factors (EMT-TFs) repress epithelial markers like E-cadherin, occludins, claudins, or ZO-1 and activate mesenchymal markers like N-cadherin, vimentin, and fibronectin. EMT is considered an essential event in dissemination of cancer clones in epithelial tumors, as breast cancer [34]. Moreover, transforming growth factor beta (TGFβ)-induced EMT has been shown to promote the dendritic cell-like migration of breast cancer cells through the lymphatic vessels [35]. Also, the co-overexpression of Oct4 and Nanog transcription factors, essential to maintain the pluripotency and self-renewal of embryonic stem cells, positively regulates the EMT process in lung adenocarcinoma (LUAD) and breast cancer, contributing to metastasis and worse prognosis [36, 37]. Oct4/Nanog co-expression is also a strong independent predictor of tumor recurrence and unfavorable outcome in hepatocellular carcinoma (HCC) by promoting EMT through activation of Stat3/Snail signaling [38]. High expression levels of activated Stat3 (p-Stat3) and VEGF are also associated with lymph node involvement in esophageal squamous cell carcinoma (ESCC), indicating that Stat3/VEGF pathway promotes cancer cell lymphatic metastasis and is correlated with tumor-node-metastasis (TNM) stage [39]. From a therapeutic standpoint, the inhibition of Stat3 has been proposed as a relevant strategy to control stem cell-associated EMT phenotype. The recent development of small molecules targeting this pathway was deeply reviewed last year [40]. The main feature of EMT induction is the loss of cell–cell adherent junctions via inhibition of E-cadherin, encoded by the *CDH1* gene. EMT inducers of the SNAIL, ZEB, and TWIST families act as direct E-cadherin repressors via binding to the E-box elements in the promoter region of the *CDH1* gene [41, 42].

Following on the metastatic cascade, the second step is the intravasation, the transendothelial migration of cancer cells into blood or lymphatic vessels through the basal lamina fenestration. Third, during circulation, cells must survive in the circulatory system and deal with coagulation, shear stress, and the velocity of blood stream mechanic. Next is the extravasation or the second site seeding, through release of TGFβ by blood platelets that enable this process by disrupting cell-to-cell endothelial junctions [43]. Finally, distant tumor cells could undergo a mesenchymal to epithelial transition (MET) to settle down at the distant microenvironment and engage proliferation and metastasis outgrowth by adaptation to the new environment. This complex process is supported by the molecular communication of the tumor cells that permit their mobility, plastic transitions, adjacent angiogenesis, and immune escape.

It is important to remark that a delay in the metastatic outgrowth of disseminated cancer cells (DCCs) that reside in metastatic niches can occur, a state known as metastatic dormancy. This phenomenon could explain the fact that a significant proportion of metastatic diseases present years-to-decades following initial diagnosis and treatment [44]. During metastatic dormancy, the tumor cells stop proliferating and stay in a quiescent state due to unfavorable proliferative conditions. This process has important clinical implications as dormant cells can be responsible of disease recurrence, as non-proliferative cells are more resistant to therapies and evade immune attacks. Neophytou and colleagues recently reviewed mechanisms that promote or impede dormancy, and how drugs could target specifically these cells [45]. Metastatic dormancy is dynamically regulated to retain their self-renewal capacity and adopt potential new phenotypes depending on the environment. Thus, dormancy requires extensive crosstalk between DCCs and the cells that comprise their microenvironments, including endothelial cells, tissue-resident and circulating immune cells, mesenchymal stem cells, and stromal fibroblasts [44]. Interestingly, epigenetic regulation by DNA methylation and histone marks has been implicated in the dormancy of the DCCs and could constitute therapeutic options [44], but with the risk of reawakening dormant cells and promoting tumor progression even after a long latency period [46]. Consequently, a deeper understanding of the interplay between dormancy and epigenetic modulations seems crucial to identify novel targets to prevent and treat metastatic disease.

## Molecular alterations in cancer metastasis

### Genetic alterations in metastasis

As previously mentioned, no specific genetic mutation seems to be determinant in metastatic progression, but some mutational combinations could enhance the metastatic ability. This topic has been reviewed few years ago by Patel et al. [8]. Although metastases have similar mutational profiles to their respective primary tumors, they commonly bear driver alterations at higher frequency than primary tumors. Priestley et al. characterized 2520 samples of metastatic tumors from 22 solid cancer types and they found no evidence of driver mutations that were specific to metastases [47], but they found that alterations in the *MLK4* gene (which encodes Mixed lineage kinase 4) are frequently associated with metastasis. Interestingly, *MLK4* upregulation has previously been linked to migratory and invasive phenotypes in breast cancer cells [48]. In addition, germline variants of *APOE* have been shown to be associated with different outcomes in melanoma. Mice expressing the human *APOE4* variant exhibited reduced metastasis compared to those expressing *APOE2*, suggesting that pre-existing hereditary genetic variants can modulate progression and clinical outcomes due to variations in metastatic competences [49]. Using mouse models, it has been shown that a combination of four mutations in genes commonly found in primary human cancers (*Apc*^*fl/fl*^, *Kras*^*LSL−G12D*^, *Tgfbr2*^*fl/fl*^, and *Trp53*^*fl/fl*^) promotes metastases, but the lack of one of them hinders the metastatic ability [50]. Besides, a study of multiregion biopsies of primary and liver metastatic regions from colorectal cancers with whole-exome sequencing and copy number profiling suggested that the genetic intratumoral heterogeneity is a potential driving force to generate metastasis-initiating clones [51]. Copy number aberrations and concomitant overexpression of *MYC*, *YAP1*, or *MMP13* also increased the incidence of brain metastasis from lung cancer [52]. This observation suggests that overexpression of genes by other mechanisms like gene-promoter hypomethylation or enhanced chromatin accessibility could result into similar outcomes. Moreover, genetic aberrations can impact central pieces of the epigenetic or epitranscriptomic machineries. To cite one example, gene amplification of the m^6^A reader YTHDF3 has been described in breast cancer brain metastases. Resultant overexpression of YTHDF3 increases translation of m^6^A-enriched transcripts as ST6GALNAC5 and GJA1, key brain metastatic genes [53].

### Epigenetic alterations in metastasis

Epigenetic modifications refer to heritable changes in gene activity that do not involve changes in the underlying DNA sequence. Epigenetic factors fine-tune gene expression programs and act as master regulators controlling essential biological functions. The main epigenetic mechanisms are DNA methylation, histone modifications, chromatin remodeling, and non-coding RNA regulation. In this section, we describe relevant epigenetic alterations involved in metastasis, summarized in Table 1.

**Table 1:** Summary of relevant epigenetic modifications implicated in cancer metastasis

Gene	Cancer type (methylation state)	Role in metastasis	Ref
DNA methylation			
S100A4, CYTIP	ccRCC (HypoM)	Promote VHL-HIF downstream gene expression for metastasis	[54, 55]
TBC1D16-47KD	Melanoma (HypoM)	Promotes metastasis by targeting RAB5C	[56]
NR2F2-Iso2	Melanoma (HypoM)	Promotes metastasis	[57]
CDH11	Melanoma (HyperM)	Represses lymph node metastases	[58]
TCF21	Melanoma (HyperM)	Represses metastasis by upregulating KISS1	[59]
TIMP3	Oral cancer, esophageal adenocarcinoma (HyperM)	Represses metastasis by regulating EMT markers through the Ras-ERK pathway	[60, 61]
TIMP3, THBS1	Gastric cancer (HyperM)	Biomarkers for peritoneal metastasis	[62, 63]
IRX1	Osteosarcoma (HypoM)	Promotes metastasis via induction of CXCL14/NF‐kB signaling	[64]
GABRP	Ovarian cancer (HypoM)	Promotes metastasis by activating the MAPK/ERK pathway	[65]
SFRP1	NSCLC (HyperM)	Represses lymph node metastasis	[66]
SFRP1	RCC (HypoM)	Promotes metastasis through MMP10-mediated pathways activation	[67]
ZNF382, GFRA1	Gastric cancer (HyperM)	Represses metastasis	[68]
CST6, BRMS1, SOX17	Breast cancer (HyperM)	Represses metastasis	[69]
CST6, ITIH5, RASSF1A	Breast cancer (HyperM)	Represses metastasis	[70]
RASSF1A	Melanoma (HyperM)	Represses metastasis	[71]
VIM, SFRP2	CRC (HyperM)	Represses metastasis by decreasing cytoskeleton formation and activating Wnt pathway	[72]
BRMS1	Breast and lung cancer (HyperM)	Represses metastasis	[73]
NME1	Breast cancer (HyperM)	Represses metastasis	[74]
KAI1	Prostate cancer (HyperM)	Represses metastasis	[75]
KAI1, NME1	Lung cancer (HyperM)	Represses distant metastasis	[76]
KISS1	ESCC (HyperM)	Represses metastasis	[77]
TET2, TET3	Melanoma (HyperM)	Repress TGFβ1-induced EMT-like process and metastasis	[78]
TET	SDH-deficient cancer cells (HyperM)	Represses EMT-like phenotype and metastasis	[79]
Nanog	Cervical cancer (HypoM)	Demethylation driven by RhoC/TET2/WDR5 that promote EMT and cancer progression	[80]
FOXO4	Gastric cancer (HypoM)	Represses Wnt/β-catenin signaling and consequently metastasis potential	[81]
CD147	NSCLC (HypoM)	Promotes metastasis by TGF-β pathway activation	[82]
miRNAs			
miR-135a	Osteosarcoma, gastric cancer	Represses pulmonary metastasis by targeting BMI1 and KLF4. Decreases metastasis. FAK pathway	[83, 84]
miR-10b	Breast cancer	Promotes metastasis by targeting HOXD10	[85]
miR-373, miR-503c	Breast cancer	Promote migration and invasion by silencing CD44	[86]
miR-335	Breast cancer	Represses migration, invasion, and colonization. SOX4, TNC	[87]
miR-126	Breast, lung, and gastric cancer	Represses migration, invasion, adhesion, and angiogenesis. VEGFA, CRK	[88–90]
miR-148a, miR-34b/c, and miR-9	Lymph node metastasis	Repress metastasis by downregulating MYC, E2F3, CDK6, and TGIF2	[91]
miR-9	Breast cancer, bladder transitional cell carcinoma	Promotes metastasis. E-cadherin, PROX1, CBX7	[92, 93]
miR-200s	Breast and colorectal cancer	Represses EMT and metastasis by targeting ZEB1/2	[94–101]
miR-22	Breast cancer	Promotes metastasis by targeting TET demethylases	[102]
miR-149	Breast cancer	Represses migration, invasion, and adhesion by targeting GIT1	[103]
miR-491-5p	Oral and breast cancer	Represses migration, invasion, and adhesion by targeting GIT1, ZNF-703, JMJD2B	[104–106]
miR-138	Ovarian, kidney, and oral cancer	Represses migration and invasion by targeting HIF1A, SOX4, RhoC, and ROCK2	[107–109]
miR-138	Cervical cancer	Represses EMT, invasion, and metastasis by targeting EZH2	[110]
miR-127	Breast cancer	Represses metastasis	[111]
miR-1306–3p	HCC	Promotes metastasis. FBXL5-Snail	[112]
miR-30d	HCC	Promotes metastasis by silencing GNAI2	[113]
miR-206	Melanoma	Represses migration by targeting CDK4 and cyclin C/D	[114]
miR-181b-3p	Breast cancer	Promotes EMT and metastasis by targeting YWHAG	[115]
lncRNAs			
PINT	Thyroid cancer	Represses metastasis by downregulating miR-767-5p and avoiding repression of TET2	[116]
ANCR	Breast cancer	Represses metastasis by promoting EZH2 degradation	[117]
PHACTR2-AS1	Breast cancer	Represses metastasis. Ribosomal genes	[118]
HOTAIR	Breast and colorectal cancer	Promotes metastasis by modulating PRC2 deposition	[119, 120]
NBAT1	Breast cancer	Represses metastasis by upregulating DKK1 via PRC2	[121]
LINC02273	Breast cancer	Promotes metastasis by regulating AGR2 expression	[122]
SChLAP1	Prostate cancer	Promotes metastasis by regulating SWI/SNF chromatin complex	[123]
LINC00978	Gastric cancer	Promotes metastasis by activating TGFβ/SMAD signaling pathway and EMT	[124]
lncRNA-ATB	Breast cancer	Promotes metastasis by competitive binding of miR-200c sites	[125]
lncRNA-CTS	Cervical cancer	Promotes metastasis and TGF-β1-induced EMT by competitive binding of miR-505 sites	[126]
Histone modifiers			
SUZ12	ccRCC	Represses metastasis via VHL-HIF downstream gene repression of CXCR4	[55]
EZH2	Various cancers	Promotes metastasis by silencing epithelial markers, TIMPs, TSGs	[127–131]
SETD2	ccRCC, LUAD	Represses metastasis by oncogenic enhancer repression, STAT1-IL-8 signaling repression	[132, 133]
SETDB1	LUAD	Represses metastasis collaborating with the SMAD2/SMAD3 repressor complex	[134]
	Breast cancer	Represses metastasis by decreasing SNAIL expression	[135]
	HCC	Promotes metastasis	[136]
DOT1L	Breast cancer	Promotes metastasis by enhancing SNAIL, ZEB1, and ZEB2 expression	[137]
PCAF	Lung cancer	Promotes metastasis by targeting EZH2	[138]
	Cancer cells and HCC	Promotes or impedes metastasis by activating or repressing EMT, respectively	[139, 140]
HDAC1, HDAC2	Pancreatic cancer	Promote metastasis by repressing E-cadherin	[141, 142]
SIRT1	Prostate cancer	ZEB1-SIRT1 silence E-cadherin, promoting metastasis	[143]
HDAC1	Breast cancer	ZNF827-HDAC1 alter the splicing of EMT regulator genes	[144]
LSD1	Breast cancer	LSD1-SNAIL silence epithelial genes promoting metastasis	[145]
KDM5B (JARID1B)	HCC	Promotes EMT and metastasis via PTEN silencing	[146]
BRD4	Melanoma	Promotes metastasis by controlling SPINK6 enhancer	[147]
	HNSCC	Promotes metastasis through super-enhancer formation at cancer stemness genes	[148]

Abbreviations: *ccRCC* clear cell renal cell carcinoma; *CRC* colorectal cancer; *EMT* epithelial to mesenchymal transition; *HCC* hepatocellular carcinoma; *HNSCC* head and neck squamous cell carcinoma; *HyperM* hypermethylated; *HypoM* hypomethylated; *lncRNAs* long non-coding RNAs; *LUAD* lung adenocarcinoma; *miRNAs* microRNAs; *TIMPs* tissue inhibitor of metalloproteinases; *TSGs* tumor suppressor genes

#### DNA methylation

DNA methylation is the most extensively studied epigenetic modification. This modification involves the addition of a methyl group to cytosine nucleotides, almost exclusively at cytosines followed by guanine (CpG sites). The methylation patterns are precisely regulated by a set of enzymes that introduce the modification through either *de novo* methylation (DNA methyltransferases DNMT3A and DNMT3B) or by full copying and preserving the methylation patterns during DNA replication (maintenance DNA methyltransferase DNMT1); and DNA demethylases (ten-eleven translocation enzymes TET1, TET2, and TET3) that actively remove the methyl group. DNA methylation can also be passively removed through sequential cell divisions in the absence of DNA methylation maintenance. Aberrant DNA methylation is a hallmark of cancer, characterized by a massive global loss of DNA methylation (hypomethylation), mainly at repetitive sequences, leading to chromosomal instability, translocations, gene disruption, and reactivation of endoparasitic sequences [149, 150]. In parallel, promoter-associated CpG island hypermethylation is a well-recognized mechanism of gene silencing of tumor suppressor genes [151, 152]. Extensive characterization of tumors has revealed metastasis-specific methylation events in several tumor types. For instance, in clear cell renal cell carcinoma (ccRCC), promoter hypomethylation-mediated increased expression of S100 calcium binding protein A4 (S100A4) and cytohesin 1 interacting protein (CYTIP) contribute to metastatic colonization [54, 55]. DNA hypomethylation enables HIF-driven CYTIP expression to protect cancer cells from death cytokine signals independently of VHL expression. Moreover, loss of the repressive histone methylation mark H3K27me3 by downregulation of the PRC2 subunit SUZ12 activates HIF-driven CXCR4 expression in support of chemotactic cell invasion [55]. In melanoma, our group has identified isoform-specific epigenetic reactivation events involved in metastasis. First, we found that DNA hypomethylation enhances the expression of a cryptic transcript of Rab GTPase‐activating protein TBC1D16 (TBC1D16-47KD), a short isoform that exacerbates melanoma growth and metastasis [56]. Interestingly, although epigenetic reactivation of TBC1D16-47KD is associated with poor clinical outcome in melanoma, it confers greater sensitivity to BRAF and MEK inhibitors, providing a potential therapeutic option [56]. More recently, we described how DNA methylation dynamically controls the expression of an alternative isoform of *NR2F2*, an orphan nuclear receptor essential for neural crest cell (NCC) development. We found that the *NR2F2*-Isoform2 (*NR2F2*-Iso2) becomes hypermethylated and silenced during NCC-to-melanocyte differentiation, but demethylation occurs in the transition from primary to metastasis in melanoma. Epigenetic reactivation of *NR2F2*-Iso2 unleashes a metastatic program in melanoma that restores the phenotypic plasticity and enables the acquisition of NCC-like features [57]. Also in melanoma, we showed that silencing of cadherin-11 (*CDH11*) occurs essentially in the lymph node metastases, suggesting a metastasis-specific role of this epigenetic event [58]. Moreover, Arab et al. found that the tumor suppressor gene *TCF21* is silenced by gene promoter hypermethylation, leading to increased risk of metastasis in melanoma. Functionally, TCF21 binds the promoter of the melanoma metastasis-suppressing gene *KISS1* and enhances its expression. Thus, epigenetic silencing of *TCF21* leads to downregulation of *KISS1* and promotes metastasis [59].

The tissue inhibitor of metalloproteinase-3 (*TIMP3*) is another example of epigenetically regulated gene involved in metastasis. Loss of *TIMP3* by promoter methylation of Sp1 binding site promotes oral cancer metastasis [60]. Interestingly, TIMP3 is able to regulate EMT by increasing the expression levels of the epithelial markers (ZO-1 and E-cadherin) and reducing the expression levels of the mesenchymal markers (vimentin, fibronectin, SNAIL, and TWIST) through the Ras-ERK pathway [60]. *TIMP3* methylation has also been implicated in the development and progression of esophageal adenocarcinoma, and reduced TIMP3 expression has been associated with increased tumor invasiveness and poor prognosis [61]. In addition, assessment of *TIMP3* hypermethylation in body fluids, including serum and preoperative peritoneal washes, has been suggested as a promising biomarker for monitoring peritoneal metastasis in gastric cancer patients after gastric surgery [62]. In the same setting, circulating methylated thrombospondin 1 (*THBS1*) has also been described as a potential biomarker for predicting peritoneal dissemination in gastric cancer [63]. Moreover, hypomethylation of iroquois homeobox 1 (*IRX1*) has been involved in osteosarcoma metastasis via induction of CXCL14/NF‐kB signaling [64]. In ovarian cancer, γ-aminobutyric acid (GABA)A receptor π subunit (GABRP) was observed upregulated first in metastatic tissues from a mouse model and after in patients due to gene promoter hypomethylation. GABRP expression was associated with ERK activation, and MAPK/ERK kinase (MEK) inhibitor U0126 could suppress the migration and invasion of cancer cells [65]. For the secreted frizzled-related protein 1 (*SFRP1*), a Wnt antagonist deregulated in several malignancies, dual roles have been described in cancer [153]. In non-small cell lung cancer (NSCLC), for instance, the epigenetic silencing of *SFRP1* is associated with lymph node metastasis and disease progression within a year after surgery [66]. In contrast, *SFRP1* is hypomethylated and upregulated in metastatic renal cell carcinoma (RCC), playing a pro-metastatic role through the activation of matrix metalloproteinase MMP10-mediated pathways [67].

Moreover, since early prediction of metastasis could improve clinical management and prognosis for early-stage patients, the use of DNA methylation biomarkers to predict metastasis has also been proposed. In gastric carcinoma, presence of methylation in *ZNF382* and *GFRA1* were found significant independent predictors of metastasis for patients with pN_0_M_0_ gastric cancer, although with a sensitivity of 61.5% and specificity of 70.1% [68]. A pilot model to predict liver metastasis in early-stage CRC patients using 23 differentially methylated regions (DMRs) has also been developed [154]. However, validations in large multi-centric cohorts are still needed to support the use of these predictors for outcome assessment in cancer patients.

Besides, the intrinsic tissue-specificity of epigenomic profiles has also been exploited to develop a DNA methylation-based predictor (EPICUP) to identify the tumor origin in metastasis of unknown primary [155]. The heterogeneous group of orphan metastatic tumors diagnosed as CUP are characterized by early dissemination, aggressive clinical course, unpredictable metastatic pattern, and dismal prognosis, with median survival of 3–6 months in more than 80% of cases [156, 157]. Considering that cancer treatment is currently largely based on the primary tumor, the lack of knowledge about the tumor origin seriously hinders clinical management and treatment decision-making in CUP patients. Thus, the use of EPICUP and other tumor-type classifiers to unmask the biological identity of CUPs are valuable tools to guide more precise therapies associated with better outcomes in CUP patients [155].

Metastases initiate with the spread from the primary or secondary tumor site to distant regions of a single cell or a cluster; thus, epigenetic modifications can also be analyzed in circulating tumor cells (CTCs), a topic recently reviewed by Vasantharajan et al. [158]. To cite some examples, Chimonidou et al. detected promoter hypermethylation of two metastasis suppressor genes (MSG) (cystatin-M-precursor (*CST6*) and breast cancer metastasis suppressor 1 (*BRMS1*)) and one tumor suppressor gene (TSG) (SRY-box transcription factor 17 (*SOX17*)) in CTCs isolated from peripheral blood of breast cancer patients. Moreover, an increase in the proportion of patients with methylated *CST6*, *BRMS1*, and *SOX17* was found from operable to metastatic breast cancer cases [69]. Later, another study validated *CST6* hypermethylation and identified two other TSGs, inter-α-trypsin heavy chain 5 (*ITIH5*) and Ras association domain family 1 isoform A (*RASSF1A*), hypermethylated in CTCs isolated from breast cancer patients when compared to the primary tumors [70]. The increased methylation of *RASSF1A* was also observed in melanoma and associated with increased metastatic potential [71]. Hypermethylation of EMT-related genes has also been detected in CTCs. For instance, in CRC, vimentin (*VIM*) and secreted frizzled-related protein 2 (*SFRP2*) were more methylated in CTCs compared to primary CRC, leading to decrease of cytoskeleton formation and increased activation of Wnt signaling pathway, respectively [72]. Downregulation of *BRMS1* through DNA methylation has also been associated with metastatic progression in TNBC [73]. The role of *BRMS1* as a metastasis suppressor has also been described in NSCLC, where DNA hypermethylation is also responsible of its epigenetic silencing. Interestingly, a study in surgically resected lung adenocarcinoma has suggested the potential of BRMS1 expression to predict future metastases. Authors have shown that low intratumoral BRMS1 expression is associated with worse overall and disease-free survival [159].

Additional examples of epigenetically regulated metastasis suppressor genes include NME/NM23 nucleoside diphosphate kinase 1 (*NME1*, alias *NM23-H1*), metastasis suppressor kangai-1 (*KAI1*), and KiSS-1 metastasis suppressor (*KISS1*). *NME1* expression is upregulated upon exposure to the DNMTi 5-Aza-2′-deoxycytidine in a dose-dependent manner in breast cancer cells disrupting its anti-metastatic properties, evidenced by a decreased cell motility [74]. Regarding *KAI1*, DNA methylation-dependent silencing of this gene has been described during prostate cancer progression [75]. Interestingly, co-downregulation of the tumor suppressor *PTEN* and the metastasis suppressors *KAI1* and *NME1* significantly correlates with distant metastasis and predicts shortened survival in NSCLC [76]. About *KISS1*, promoter hypermethylation partly contributes to its downregulation in ESCC. In this tumor type, *KISS1* inhibits the metastasis by targeting the MMP2/9/ERK/p38 MAPK axis [77].

Opposite to the role of DNMTs, the TET family of methylcytosine dioxygenases demethylate cytosine nucleotides (5mC) through a succession of oxidization to 5-hydroxymethylcytosine (5hmC), 5-formylcytosine (5fC), and 5-carboxylcytosine (5caC) [160]. Of note, TETs and other α-ketoglutarate-dependent dioxygenases like histone demethylases and ALKBH demethylases are sensitive to the increase of succinate, fumarate, and D-2-hydroxyglutarate that accumulate when SDH, FH, and IDH1/2 enzymes are mutated, respectively [161, 162]. This inhibition of activity leads to a specific hypermethylation phenotype named CpG island methylator phenotype (CIMP), characterized by the concerted hypermethylation of a large number of genes that can drive tumor progression [163–165]. CIMP-high (hypermethylation) has been associated with metastasis in oral cancer [166], CRC [167], HCC [168], melanoma [169], esophageal squamous cell carcinoma [170], gastric cancer [171], papillary thyroid carcinoma [172], and inversely associated in breast cancer [173]. In prostate cancer, BAZ2A (TIP5) was found overexpressed and interacting with EZH2 to maintain silenced anti-metastatic genes [174]. Of note, DNA methylation-induced silencing of TET2 and TET3 in melanoma was shown to promote a TGFβ1-induced EMT-like process and metastasis [78]. Regarding EMT, it has shown by Morin et al. that SDH-deficient cells undergo TET silencing and hypermethylation of PRC2 target genes, but cells required additional HIF-2α activation for driving EMT-like phenotype and metastasis [79]. In melanoma, global hydroxymethylation of DNA cytosine nucleotides, driven by TET activity, was observed decreased and low 5hmC level was associated with the presence of metastasis. Interestingly, TET1 level was decreased in WNT subgroup while TET3 level was decreased in SHH subgroup with worse prognosis [175]. In cervical cancer, PRMT5 (protein arginine N-methyltransferase 5) forms a complex with Snail, HDAC1/2, MTA1, Mep50, and NuRD, and represses the expression of TET1 and E-cadherin by histone methylation on their promoter regions, leading to EMT initiation and increased risk of metastasis [176]. Also in this cancer type, Thomas et al. have shown that the pro-metastatic protein RhoC associates with TET2 and WDR5 to drive demethylation of some pluripotency genes such as *Nanog* to drive EMT and cancer progression [80]. In gastric cancer metastasis, the expression of TET1 was shown to be reduced and predicted poor survival. The authors have shown that TET1 targets *FOXO4* gene-promoter methylation, leading to increased FOXO4 expression that negatively regulates Wnt/β-catenin signaling and consequently represses metastasis potential [81]. Concerning upregulation of TET level associated with metastasis, it was shown in NSCLC that CD147 expression is controlled by active demethylation of its promoter, leading to increased expression comparing with normal tissues. Treatment with TGFβ triggers active demethylation of *CD147* by the loss of the KLF6/MeCP2/DNMT3A complex and recruitment of Sp1, TET1, TDG, and SMAD2/3 to the *CD147* promoter [82]. Interestingly, authors successfully inhibited invasion and metastasis in NSCLC mouse models by using a targeted methylation system to silence CD147 expression [82], providing a promising therapeutic target for NSCLC. An example involving various epigenetic regulations is depicted in thyroid cancer where long non-coding RNA (lncRNA) PINT is downregulated, leading to upregulation of the microRNA miR-767-5p and repression of TET2. This regulation was significantly associated with advanced TNM stage and lymph node metastasis [116]. These selected examples demonstrate how cancer cells regulate pro-metastatic gene expression by gene-promoter demethylation to enhance metastasis potential. Regarding factors involved in stemness-like features and EMT, it has been shown that Nanog expression, controlled by OCT3/4 and SOX2, can be regulated by DNA methylation and correlates with different states of germ cell tumors [177].

#### Non-coding RNAs

Different players of the epigenetic machinery can interplay to generate a meaningful biological outcome. For instance, some microRNAs (miRNAs), small non-coding RNAs that regulate gene expression by binding to the 3′-untranslated regions (3′-UTRs) of specific mRNAs [178], directly repress enzymes of the epigenetic machinery, including DNA methyltransferases (DNMTs), histone deacetylases (HDACs), and histone methyltransferases (HMTs). Few years ago, Liu et al. have shown that miR-135a combined with SMYD4 activates Nanog expression inducing the switch of non-cancer stem cells into cancer stem cells [179]. They described that miR-135a lowers the methylation of *Nanog* promoter by targeting DNMT1 [179]. Of note, although miR-135a mostly promotes cellular proliferation and cancer progression, it can play opposite roles depending on the cancer type [180]. For example, miR-135a has been described as a tumor suppressor in osteosarcoma pulmonary metastasis by targeting BMI1 and KLF4 [83], and in gastric cancer by downregulating focal adhesion kinase (FAK) pathway, suggesting FAK inhibition as a potential therapeutic strategy [84].

On the other hand, DNA methylation can also regulate the expression of non-coding transcripts, including miRNAs. In gastric cancer, it was shown that miR-10b is silenced by gene promoter methylation and it leads to increased expression of the oncogene microtubule-associated protein, RP/EB family, member 1 (MAPRE1), meaning a tumor suppressive role in this context [181]. Strikingly, a pivotal study in the field of miRNAs involved in metastasis described a pro-metastatic role of miR-10b in breast cancer. Mechanistically, Twist induces miR-10b expression, which directly targets and inhibits HOXD10, resulting in increased expression of the pro-metastatic gene *RHOC* [85]. Also in breast cancer, miR-373 and miR-520c promote metastasis through suppression of CD44 [86]. Other examples of epigenetically regulated miRNAs include miR-126 in CRC and cancer cell lines, where it was shown that inhibitors of DNA methylation and histone deacetylation can restore its expression [182, 183], and miR-335 in breast cancer [87]. Of note, miR-126 was found downregulated in breast, gastric, and lung cancer, promoting metastasis [88–90]. Our group also described a miRNA DNA methylation signature for metastasis that involves the epigenetic silencing of miR-148a, miR-34b/c, and miR-9, mediating the upregulation of oncogenic target genes such as MYC, E2F3, CDK6, and TGIF2 [91]. Of note, expression of these miRNAs could be restored by treatment with DNA-demethylating agent. Regarding miR-9, although it was found hypermethylated in metastases, and metastasis suppressor features were revealed in *in vitro* and *in vivo* assays [91], a pro-metastatic role has been described in other studies, suggesting a context-dependent function. In breast cancer, miR-9 increases cell mobility and invasiveness by directly targeting E-cadherin [92]. In CRC cells, miR-9 is regulated by PROX1 that bound its promoter and trigger its expression to suppress E-cadherin and promote EMT [184]. In bladder transitional cell carcinoma, the CBX7 is another target silenced by miR-9 [93], and it has been shown that CBX7 suppresses metastasis of basal-like breast cancer through Twist1/EphA2 pathway [185].

Thus, the induction of EMT is accompanied by a dynamic reprogramming of the epigenome involving changes in DNA methylation and several post-translational histone modifications, not only in protein-coding but also in non-coding genes [186, 187]. A family of miRNAs particularly relevant in EMT and metastasis is miR-200, considering its role as a master regulator of the epithelial phenotype by targeting ZEB1 and ZEB2, transcriptional repressors of E-cadherin [94]. Our group and others described that the miR-200b/200a/429 (miR-200ba429) and miR-200c141 polycistronic transcripts (miR-200s) undergo hypermethylation-associated silencing linked to EMT in tumor progression and metastasis in several tumor types [95–98]. Moreover, we have shown that the epigenetic regulation of miR-200s is a dynamic process that mediates the shifts between EMT and MET phenotypes [95]. Considering the aberrant expression in various cancers, miR-200 family members have been proposed as diagnostic and prognostic biomarkers, including the assessment of the circulating miRNAs in serum as a non-invasive approach [99]. In addition, the epigenetic regulation of this family led to the hypothesis that epigenetic drugs like HDAC inhibitors could restore miR-200s expression and constitute a potential therapeutic strategy in triple-negative breast cancer [99–101]. Importantly and in the same line, it was observed that miR-22 can increase metastasis potential of breast cancer cells by silencing the anti-metastatic miR-200 via targeting of the TET (ten-eleven translocation) family of methylcytosine dioxygenases, thereby inhibiting demethylation of the miR-200 promoter [102].

Interesting examples include also miR-149. In gastric cancer, the epigenetic silencing of miR-149 has been observed in cancer-associated fibroblasts (CAF), where the miR-149 links PGE2 and IL-6 signaling to mediate the crosstalk between tumor cells and CAFs [188]. Particularly, miR-149 inhibits fibroblast activation by targeting IL-6. In glioblastoma, miR-149 was shown epigenetically silenced and its restoration suppressed the expression of AKT1 and cyclin D1 and reduced the proliferative activities of glioma cells, without being correlated with metastasis potential [189]. In breast cancer, miR-491-5p gene is hypermethylated, leading to decreased expression. miR-491-5p directly targets JMJD2B/KDM4B, a histone demethylase with oncogenic features, suggesting a possible downstream epigenetic-mediated tumor suppressor effect [104]. In breast cancer, miR-149 also acts as a suppressor of metastasis by targeting GIT ArfGAP 1 (GIT1) to inhibit integrin signaling [103]. In oral squamous cell carcinoma (OSCC), miR-491-5p decreased expression was observed in invasive cells and it was also shown to target GIT1 [105]. miR-491-5p overexpression reduced GIT1 expression and inhibited migration and invasion of OSCC cells and GIT1 overexpression was sufficient to invert this process [105]. In breast cancer, miR-491-5p suppresses metastasis through ZNF-703 to regulate AKT/mTOR pathway, suggesting miR-491-5p and ZNF-703 as potential therapeutic targets for this tumor type [106]. miR-138 has also been found dysregulated in various tumor types, notably RCC [107], ovarian cancer [108], and oral cancer [109]; targeting HIF-1α, SOX2 and HIF-1α, or RhoC and ROCK2, respectively. miR-138 can also inhibit MYC expression and suppress tumor growth of CRC and HCC cell lines [190]. Interestingly, intravenous administration of miR-138 significantly impedes MYC-driven tumor growth *in vivo* [190]. In cervical cancer, CpG hypermethylation-associated decreased expression of miR-138 has been detected in tumors from patients with lymph node positivity in comparison with the non-metastatic tumor tissues [110]. Interestingly, the authors demonstrated that miR-138 suppresses tumor progression by targeting the histone methyltransferase EZH2 [110]. miR-127 is downregulated in breast cancer by Tudor-SN protein and contributes to metastasis [111], although treatment with DNA-demethylating agents and HDAC inhibitors is able to induce miR-127 expression in T24 cells, suggesting epigenetic regulation as a possibly mechanism in other cancer types [191]. In HCC, miR-1306–3p plays a pro-metastatic role where it was found upregulated and targeting FBXL5 and consequently reduce Snail degradation [112], and miR-30d promotes tumor metastasis by silencing Galphai2 (GNAI2) [113].miRNAs can also influence metastasis by targeting cell cycle-related genes. Indeed, miR-206 has a metastasis suppressor activity by targeting CDK4 and cyclin C/D in melanoma [114]. A functional role of miR-206 has been shown in HeLa cells where miR-206 acts as pro-apoptotic activator by directly targeting *Notch3* impairing tumor formation [192]. A role in EMT and breast cancer metastasis has been described for miR-181b-3p by downregulating YWHAG, which induces Snail stabilization and EMT phenotype [115]. Of note, YWHAG has been shown upregulated and promoting gastric cancer progression through EMT [193], and the YWHAG inhibitor curcumenol in combination with cisplatin has been recently proposed as a therapeutic strategy in gastric cancer [194]. Altogether, these examples highlight the key role of miRNA in controlling EMT and metastasis, by targeting critical genes involved in tumor progression and invasiveness. However, further studies are encouraged to unravel the molecular mechanisms behind miRNA and metastasis correlative data.

Another type of non-coding RNAs with important functions in regulating gene expression in metastasis is the long non-coding RNAs (lncRNAs). This topic has been largely reviewed [195–199] and only some examples are described below. The lncRNAs can play different regulatory roles as they can serve as guides for epi-regulators, change chromatin architecture, or have enhancer-like function (lncRNAs called eRNAs) [195]. In breast cancer, the lncRNA ANCR decreases metastasis by facilitating the degradation of EZH2, the catalytic subunit of the polycomb repressive complex 2 (PRC2). ANCR potentiates the interaction between CDK1 and EZH2, increasing EZH2 phosphorylation and degradation by ubiquitination. ANCR was observed downregulated in breast cancer, and its overexpression reduced lung metastases in a mouse model [117]. Interestingly, EZH2 can also target a lncRNA, PHACTR2-AS1, that represses ribosomal gene expression. The authors observed that EZH2 silenced PHACTR2-AS1 and it led to instability of ribosomal DNA, which promoted cancer cell proliferation and metastasis in breast cancer [118]. Apart direct interactions with proteins or genes, lncRNAs can also modulate chromatin conformation. This is the case for HOTAIR that is overexpressed in primary breast cancer and metastasis. Its increased expression induced genome-wide re-targeting of PRC2 to a pattern resembling more embryonic fibroblasts. This PRC2 dysregulation led to altered H3K27 methylation and gene expression, driving cancer invasiveness and metastasis [119]. Importantly, these results were later validated in CRC, further supporting the role of this lncRNA in metastasis [120].

Another example is NBAT1, a lncRNA with metastasis suppressive functions, downregulated in breast cancer and associated with poor survival and increased risk of metastasis. NBAT1 increases the expression of DKK1, an inhibitor of Wnt signaling pathway, by suppressing EZH2-induced H3K27me3 of DKK1 [121]. Another example of gene promoter regulation by lncRNA in this tumor type is the increased expression of LINC02273, stabilized by hnRNPL protein. This complex was shown to increase the levels of two histone modifications associated with active expression (H3K4 trimethylation and H3K27 acetylation) at the AGR2 gene promoter, leading to AGR2 upregulation [122]. In prostate cancer, Prensner et al. have found that SChLAP1 lncRNA also regulates the SWI/SNF chromatin modifying complex. SchLAP1 antagonizes the genome-wide localization and the tumor suppressor regulatory function of the SWI/SNF complex, increasing cancer cell invasion and metastatic spread [123]. Regarding EMT, Fu et al. have found in gastric cancer that lncRNA LINC00978 expression is elevated and correlates with tumor size, lymphatic metastasis, and TNM stage. LINC00978 activates the TGF-β/SMAD pathway and EMT by increasing the expression of Twist1 and Slug (Snail2), leading to the upregulation of N-cadherin and vimentin, but the downregulation of E-cadherin in gastric cancer cells [124]. Interestingly, lncRNAs can also regulate some miRNAs to promote metastasis. For example, lncRNA-ATB in breast cancer promotes metastasis and tastuzumab resistance by competitively binding miR-200c sites, upregulating ZEB1 and ZNF-217, and then inducing EMT [125], and lncRNA-CTS in cervical cancer promotes metastasis and TGFβ1-induced EMT by competitive binding of miR-505 sites, consequently upregulating ZEB2 [126]. Many other lncRNAs regulate metastasis through different pathways. A recent review from Ming et al. is recommended for further details [196].

#### Histone modifications

**Fig. 2:**
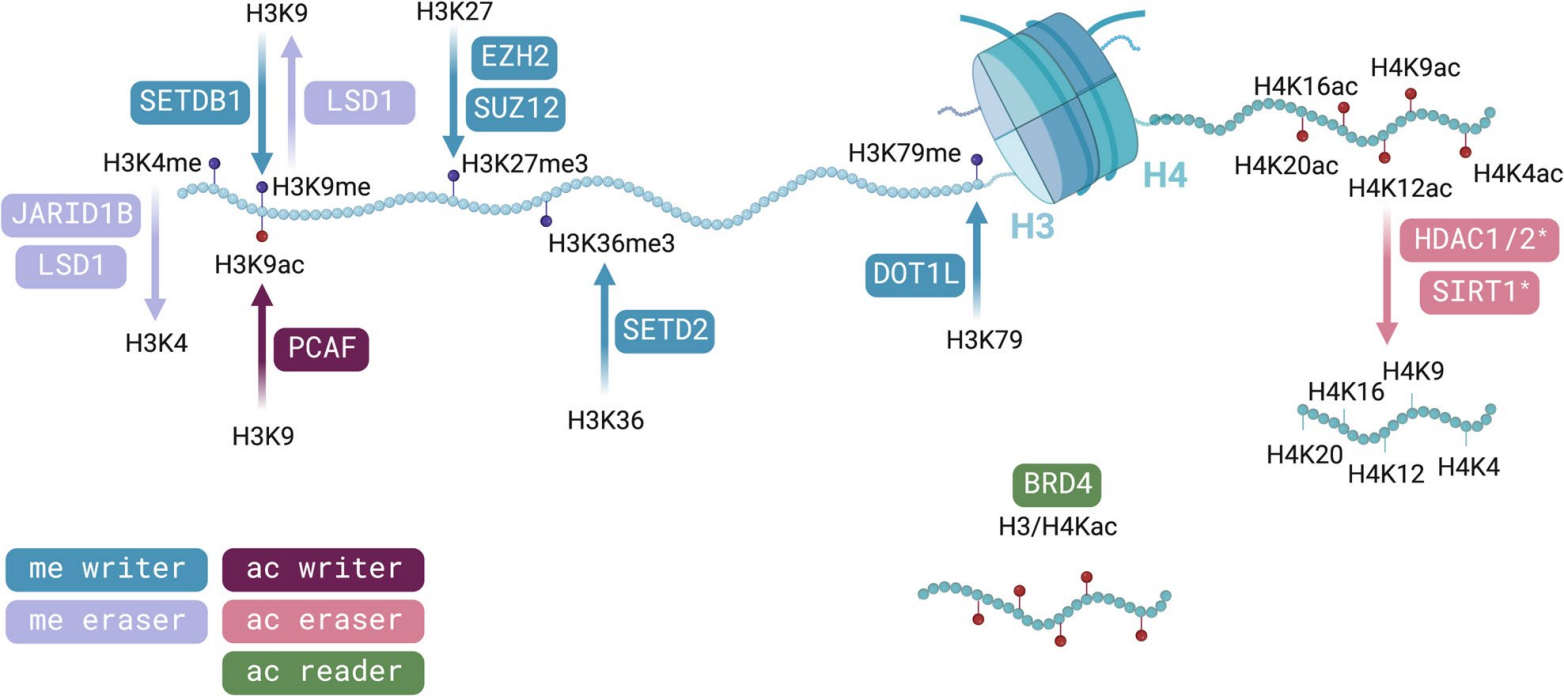
Relevant histone modifiers implicated in metastasis. Note that only histone modifiers discussed in this review are depicted, including enzymes that introduce (writers), recognize (readers), and remove (erasers) epigenetic marks to the histone tails. Abbreviations: ac, acetylation; H3, histone 3; H4, histone 4; K, lysine; me, methylation; me3, trimethylation. *HDACs also deacetylate other histones and non-histone proteins. Created with BioRender.com

Another epigenetic mechanism driving gene expression is through histone modifications (Fig. 2). The most common histone post-translational modifications (PTMs) are methylation, acetylation, ubiquitination, and phosphorylation. The crosstalk among the different marks configures the so-called *histone code* that dictates the chromatin structure in which DNA is packaged and can orchestrate the ordered recruitment of enzyme complexes to wrap the DNA. This histone code is *written* by histone-modifying enzymes that catalyze the introduction of chemical modifications in a residue-specific manner (e.g., histone lysine methyltransferases or histone lysine acetyltransferases), and *erased* by enzymes that remove the marks (e.g., histone lysine demethylases or histone lysine deacetylases). This code is interpreted by *reader* or effector proteins that specifically bind to a certain type, or a combination, of histone modifications and translate the histone code into a meaningful biological outcome, whether it is transcriptional activation or silencing, or other cellular responses [200]. In addition to this recruitment mechanism, histone marks can modulate the chromatin conformation per se based on steric or charge interactions. For instance, neutralization of the positive charges of histones by acetylation of lysines weakens the histone tail—DNA interactions that lead to chromatin decompaction, which facilitates DNA accessibility [200, 201].

Disturbance of the histone code leads to deregulated gene expression and perturbation of cellular identity, and is therefore a major contributor to cancer initiation, progression, and metastasis. Miswriting, misinterpretation, and mis-erasing of histone modifications are linked to tumorigenesis [201–203]. Moreover, EMT transcription factors, such as SNAIL, SLUG, TWIST, ZEB1, and ZEB2, recruit histone-modifying complexes to chromatin to mediate epigenetic silencing of genes involved in metastasis [187, 204]. During EMT, together with DNA methylation, histone modifications orchestrate the repression or activation between epithelial and mesenchymal genes.

Deregulated expression of histone modifiers is a common event in cancer. To cite some examples involved in metastasis (Table 1), the histone methyltransferases (HMTs) enhancer of zeste homolog 2 (EZH2), SET domain containing 2 (SETD2), SET domain bifurcated histone lysine methyltransferase 1 (SETDB1), and DOT1 like histone lysine methyltransferase (DOT1L); the histone demethylases (HDMs) lysine demethylases 1A (KDM1A/LSD1) and 5B (KDM5B/JARID1B); the histone acetyltransferases (HATs) CREB1-binding protein (CBP)/p300 and p300-CBP-associated factor (PCAF); and histone deacetylases (HDACs) (Fig. 2).

EZH2, the enzymatic subunit of PRC2 that trimethylates histone H3 lysine 27 (H3K27me3) to promote transcriptional silencing, is aberrantly expressed in cancer. Overexpression of EZH2 is a marker of advanced and metastatic disease in many solid tumors, including prostate and breast cancer [127]. EZH2-mediated silencing of several genes through H3K27me3 (e.g., E-cadherin and tissue inhibitors of metalloproteinases) favors cell invasion and metastatic spreading [128–130]. EZH2 also negatively regulates the transcription of the metastasis suppressor gene *RKIP* in breast and prostate cancer by regulating H3K27 and H3K9-me3 modifications, leading to negative association of EZH2 expression with relapse-free survival in breast cancer [131]. EZH2 can also induce the gene silencing of tumor suppressors like DAB2IP to regulate EMT and metastasis in CRC [205]. EZH2 inhibitors could be interesting as a potential treatment to reduce metastasis potential in these cases. Remarkably, in addition to the classical repressor role of EZH2 by trimethylating H3K27, EZH2 is able to methylate non-histone substrates, such as JARID2, STAT3, GATA4, FOXA1, RORα, or PLZF, to regulate their transcriptional activities or protein stability [206]. Related to metastasis, for instance, EZH2-dependent methylation of p38α enhances its stability and function; and the cooperation between EZH2 lysine methyltransferase and p38 kinase activities promotes breast cancer growth and metastasis. Interestingly, the dual inhibition of EZH2 and p38 decreases cancer progression in mouse models of TNBC [207]. Besides, EZH2 also performs relevant methyltransferase-independent functions, and it can act as transcriptional co-activator by forming complexes with other proteins or acts as a transcription factor via direct binding to the target gene promoters. To cite some metastasis-related examples of non-canonical functions of EZH2 beyond the polycomb complex, EZH2 promotes the transcriptional activation of the non-canonical NF-κB subunit RelB to drive self-renewal contributing to the maintenance of TNBC tumor initiating cells (TICs), cancer stem cells involved in tumor recurrence and metastasis [208]. Moreover, a recent study has revealed the cooperation between EZH2 and TGFβ signaling in promoting bone metastasis of breast cancer also through a methyltransferase-independent manner [209]. Mechanistically, EZH2 acts as a transcriptional co-factor of RNA Pol II to facilitate its binding to the *ITGB1* promoter, triggering its transcription. Increased ITGB1 activates FAK that phosphorylates TGFβRI and enhances its binding to TGFβRII, thereby activating TGFβ signaling [209]. This finding has critical clinical implications, underlining that targeting EZH2 methyltransferase activity by EZH2 inhibitors might not yield inhibitory efficacies in this setting, whereas targeting the downstream effector FAK with a clinically applicable kinase inhibitor could block EZH2-induced breast cancer bone metastasis, as was shown in mouse models [209]. Thus, EZH2 behaves as a bifunctional molecule that could act as a transcriptional inhibitor or a transcriptional activator in a context-dependent manner. Altogether, the interplay between the EZH2 PRC2-dependent and EZH2 PRC2-independent functions contributes to its overall impact on metastasis. Through these mechanisms, EZH2 can regulate the expression of genes involved in metastasis, such as those associated with cell adhesion, migration, invasion, and angiogenesis.

Regarding SETD2, the HMT responsible of trimethylating H3K36, it is mutated in a range of tumor types, including 13% of cases in ccRCC [210, 211]. Mechanistically, a study in ccRCC has described that the loss of SETD2-mediated H3K36me3 activates enhancers to drive oncogenic transcriptional output through regulation of chromatin accessibility [132]. In LUAD, SETD2 inhibits metastasis via regulation of STAT1-IL-8 signaling-mediated EMT [133]. Other SET domain containing protein, SETDB1, which mediates the incorporation of the repressive H3K9me3 mark and inhibits gene transcription, suppresses EMT and controls cancer cell motility, invasion, and metastatic dissemination. SETDB1 cooperates with SMAD2/SMAD3 repressor complex in TGFβ-mediated lung cancer metastasis [134]. Also, SMAD3-mediated recruitment of SETDB1 to the SNAI1 promoter region to decrease Snail1 expression and EMT has been described in breast cancer [135]. In hepatocellular carcinoma, conversely, SETDB1 activating mechanisms at the chromosomal (copy gain at 1q21), transcriptional, and post-transcriptional levels result in SETDB1 upregulation that promotes metastasis [136]. About the H3K79 methyltransferase DOT1L, an example that clearly illustrates the crosstalk between several epigenetic players to activate EMT regulators has been described in breast cancer. H3K79me-induced and H3ac-induced epigenetic derepression of SNAIL, ZEB1, and ZEB2 is accomplished by the recruitment of the HMT DOT1L and the HAT CBP/p300 to the E-box regions of gene promoters together with c-Myc. Moreover, DOT1L enhanced activity in human breast cancer leads to dissociation of HDAC1 and DNMT1 proteins from promoters, which inhibits HDAC activity and DNA methylation. The resulting epigenetic activation of EMT transcription factors promotes EMT-induced cancer stem cell properties and enhances the invasive and metastatic abilities in breast cancer, possibly reversed by the use of DOT1L inhibitor [137].

Epigenetic reactivation of metastasis-promoting genes also involves histone acetylation mediated by other HATs as PCAF. Besides, other than its HAT activity, PCAF is able to introduce post-transcriptional modifications to other substrates. For instance, acetylation of EZH2 at K348 by PCAF regulates its stability and promotes lung cancer cell migration and invasion [138]. PCAF-mediated acetylation of the transcription factor ISX promotes translocation of ISX-BRD4 to the nucleus, where the ISX–BRD4 complex unpacks chromatin and activates the expression of EMT regulators through acetylation of histone H3, subsequently promoting EMT and metastasis [139]. However, it has been described that PCAF suppresses metastasis and EMT in HCC by targeting the EMT regulator Gli1 [140].

Regarding HDACs, considering their crucial role of removing acetyl groups from lysine residues to drive gene silencing, dysregulation of HDACs disrupts gene expression and is involved in disease. Several examples that demonstrate the role of HDACs in metastasis have been described. Class I HDACs (HDAC1, 2, 3, and 8) are often overexpressed in cancer. EMT-related factors form transcriptional repressor complexes with HDACs to downregulate E-cadherin. For instance, HDAC1 and HDAC2 are recruited to the E-cadherin promoter by ZEB1/2 or SNAIL in pancreatic cancer [141, 142]. ZEB1 can also recruit the class III HDAC SIRT1 to silence E-cadherin and promote EMT and metastasis in prostate cancer cells, and the authors suggested the use of SIRT1 inhibitor as therapeutic strategy against metastasis cascade [143]. Another intriguing mechanism promoting EMT in brain development and breast cancer metastasis involves the recruitment of HDAC1 by the zinc finger protein ZNF827 that slows RNA polymerase II progression and alters the splicing of genes encoding key EMT regulators [144]. On the other hand, EMT transcription factors also recruit HDMs to orchestrate the remodeling of the transcriptomic landscape. SNAIL, for instance, recruit LSD1 to epithelial gene promoters, leading to H3K4me2 demethylation and subsequent silencing of target genes to enhance tumor metastasis [145]. Other HDMs as KDM5B/JARID1B promote EMT and metastasis in HCC cells through modulation of H3K4me3 at the PTEN gene promoter, inactivating PTEN transcription. Importantly, PI3K and AKT inhibitors could benefit to these patients [146].

As previously commented, epigenetic mechanisms, including histone modifications, are also key to support the phenotypic plasticity of disseminated cancer cells within metastatic niches [44]. Recent studies have shown that chromatin remodeling and transcriptional reprogramming control onset and escape from dormancy. To cite a key example, the retinoid-responsive gene *NR2F1* has been identified as a master regulator of tumor cell dormancy. NR2F1 induces not only a global chromatin-repressive state, but also local active chromatin changes in its own promoter and *SOX9* and *RARβ* promoters [212]. Importantly, an agonist of NR2F1 that specifically activates dormancy programs in malignant cells has been recently described [213]. More relevant, the treatment with this agonist resulted in inhibition of lung metastasis in head and neck squamous cell carcinoma (HNSCC) mouse models, supporting the use of NR2F1 agonists to induce dormancy as a therapeutic strategy to prevent metastasis [213]. In addition, combined use of DNA-demethylating agent (5-azacytidine, AZA) and activation of retinoic acid signaling (all-trans retinoic acid, atRA) is sufficient to recapitulate the quiescence program and induce chromatin changes linked to a durable dormant state. Recent data suggest that AZA + atRA reprogramming therapy is able to suppress metastasis via induction of a dormancy-like program by TGFβ-SMAD4-dependent mechanisms (NR2F1 independent or NR2F1 complementary) [214]. Further studies in this field are guaranteed to exploit the therapeutic opportunities in the clinical setting.

An additional layer of complexity is defined by the *readers* or effector proteins that recognize histone modifications. Among them, the BET family of chromatin *readers* (BRD2, BRD3, BRD4, and BRDT) contains a bromodomain that recognizes acetylated lysine residues in histones H3 and H4, triggering chromatin remodeling and transcriptional activation via recruitment of other proteins. BET proteins act as key regulators of oncogene expression by controlling super-enhancers that regulate critical oncogenic drivers, including *MYC* [215]. In melanoma, BRD4 directly interacts with the enhancer of SPINK6 and mediates its expression. SPINK6 plays an important role in melanoma migration, invasion, and metastasis. SPINK6 bounds and activates EGFR leading to an interplay between EGFR and EphA2 that caused increased phosphorylation of both receptors as well as AKT and ERK activation, promoting metastasis. Of note, BRD4 inhibitor NHWD-870 strongly reduced the invasion and metastasis of melanoma both *in vivo* and *in vitro* [147]. In HNSCC, BRD4 recruits mediators and NF-κβ p65 to form super-enhancers at cancer stemness genes such as *TP63*, *MET*, and *FOSL1*, instead of *MYC*. Interestingly, the use of BET inhibitors disrupts the super-enhancers, eliminates cancer stem cells, and inhibits HNSCC invasive growth and metastasis [148]. As discussed at the end of this review, development of *epidrugs*, drugs that target enzymes involved in epigenetic regulation of genome function, is an active field of research as a strategy for tackling cancer.

### Epitranscriptomic alterations in metastasis

**Fig. 3:**
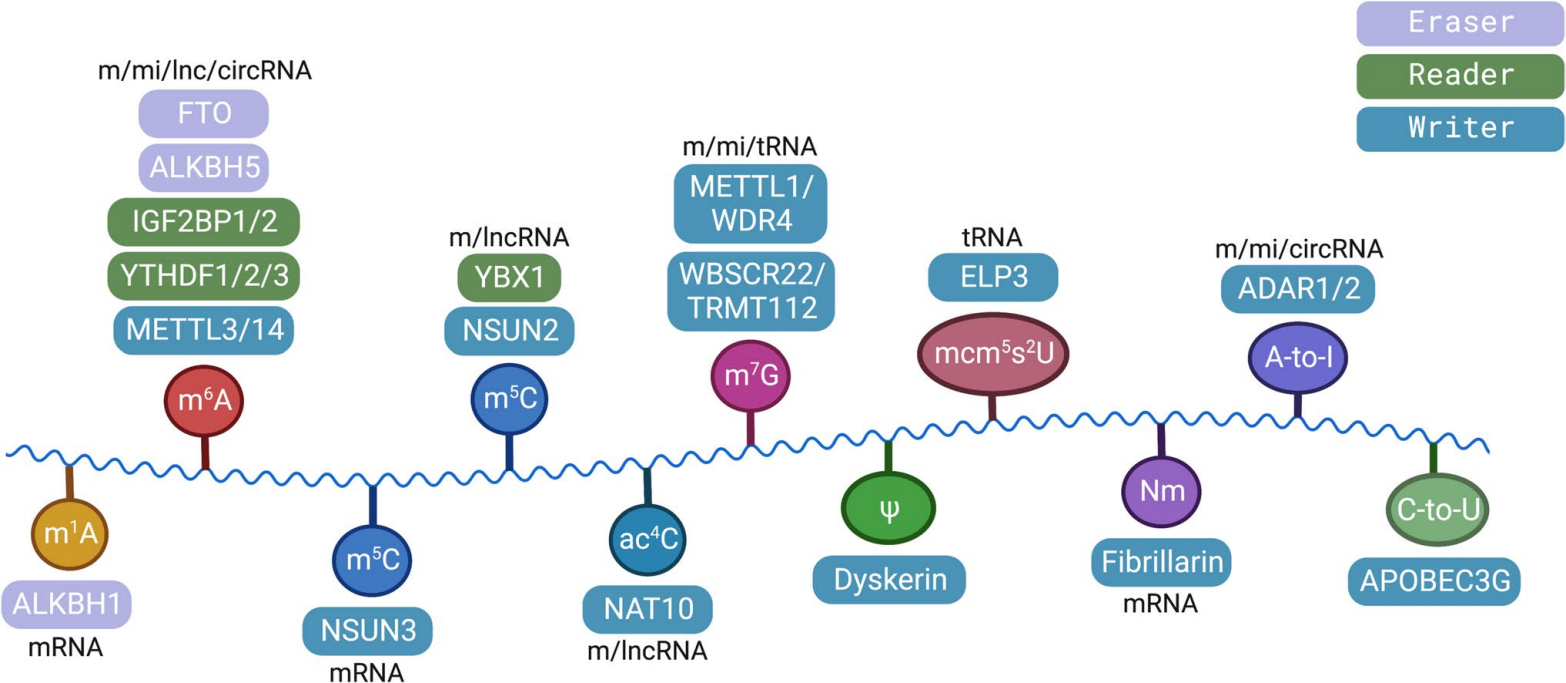
Relevant RNA modifiers involved in metastasis. Note that only RNA modifiers discussed in this review are depicted, including enzymes that introduce (writers), recognize (readers), and remove (erasers) the epitranscriptomic marks. The different RNA modifications are shown (m^1^A, N1-methyladenosine; m^6^A, N6-methyladenosine; m^5^C, 5-methylcytosine; ac^4^C, N4-acetylcytidine; m^7^G, N7-methylguanosine; Ψ, pseudouridine; mcm^5^s^2^U, 5-methoxycarbonylmethyl-2-thiouridine; Nm, 2′-O-methylation; A-to-I, adenosine-to-inosine editing, C-to-U, cytidine-to-uridine editing) with the implicated RNA modifiers and the type of RNA molecules targeted in metastasis: messenger RNA (m), microRNA (mi), long non-coding RNA (lnc), circular RNA (circ), and transfer RNA (tRNA). Created with BioRender.com

Chemical modifications deposited in RNA molecules shape another layer of biological complexity, the epitranscriptome. More than 170 modifications have been identified [216], most of them originally described in highly abundant non-coding RNAs, including ribosomal RNAs (rRNAs), transfer RNAs (tRNAs), and small nuclear RNA (snRNAs), but later also found in messenger RNAs (mRNAs). The functional significance of RNA modifications has begun to be uncovered in the last few years, and increasing evidences are supporting their role in diseases, including cancer [217]. In this section, we describe epitranscriptomic events particularly related to metastasis, summarized in Table 2 and depicted in Fig. 3. Moreover, considering the interplay between molecular mechanisms controlling gene expression, we highlight those targets impacted by both epitranscriptomic and epigenetic modifications. Also, epitranscriptomic modifications can regulate epigenetic modifiers like microRNAs or lncRNAs that will regulate downstream gene expression, a topic recently reviewed by Bove et al. for m^6^A modification [218].

**Table 2:** Summary of relevant RNA modifications implicated in cancer metastasis

RNA modifiers (function)^a^	Target (effect on the target)^b^	Role in cancer	Ref
m^6^A			
METTL3 (w)	miR-1246 (up), SPRED2 mRNA (down)	Promotes metastasis in CRC	[219, 220]
	KRT7-AS mRNA (up)	Promotes lung metastasis in breast cancer, induction of EMT	[221]
	lncRP11 (up)	Promotes liver metastasis in CRC by increasing Siah1 and Fbxo45 mRNA degradation and preventing ZEB1 mRNA decay	[222]
	HDGF mRNA (up)	Promotes liver metastasis in gastric cancer	[223]
	STEAP2 mRNA (up)	Decreases PTC progression	[224]
	UCK2 mRNA (up)	Promotes metastasis in melanoma by enhancing the Wnt/β-catenin pathway	[225]
	MALAT1 lncRNA (up)	Promotes metastasis in breast cancer	[226]
	miR-143-3p (up)	Promotes brain metastasis by silencing VASH1 in lung cancer	[227]
METTL3 (w) IGF2BP2 (r)	SOX2 mRNA (up)	Promotes stem cell phenotype and metastasis in breast cancer Promotes metastasis in CRC	[228, 229]
METTL3 (w) YTHDF1 (r)	HK2 mRNA (up)	Promotes cervical cancer	[230]
	SNAI1 mRNA (up)	Promotes EMT in cancer cells	[231]
METTL3 (w) YTHDF2 (r)	SOCS2 mRNA (down)	Promotes lung metastasis in HCC	[232]
	PTEN mRNA (down)	Promotes metastasis in gastric cancer. Linc00470-METTL3	[233]
	miR-1915-3p (down)	Promotes NSCLC migration and invasion	[234]
	RRAS, SETD7, KLF4 mRNAs (down)	Promote bladder cancer	[235, 236]
	YPEL5, USP4, DUSP5, IFFO1 mRNAs (down)	Promotes metastasis in colon, prostate, gallbladder, and other cancer types	[237–240]
METTL3 (w) YTHDF3 (r)	EGR1 mRNA (up)	Promotes EMT and metastasis in ESCC by increasing activation of EGR1/SNAIL signaling	[241]
METTL14 (w) YTHDF2 (r)	NEAT1 lncRNA (down)	Represses metastasis in RCC	[242]
	ITGB4 mRNA (down)	Represses metastasis in RCC	[243]
	SOX4 mRNA (down) XIST lncRNA (down)	Represses metastasis in CRC	[244, 245]
YTHDF1 (r)	eIF3C mRNA (up)	Promotes metastasis in ovarian cancer	[246]
	EGFR, ATG2A, ATG14 mRNAs (up)	Promotes metastasis in HCC	[247, 248]
	USP14, ARHGEF2, FOXM1, PKM2, PLK1, SLP2, ANLN, DDX23, HRAS mRNAs (up)	Promotes metastasis in gastric, colorectal, breast, prostate, liver, pancreas, and other cancer types	[249–257]
YTHDF2 (r)	OCT4 mRNA (up)	Promotes lung metastasis in HCC	[258]
	FYN mRNA (up)	Promotes metastasis in HCC with PA2G4 upregulation	[259]
	AXIN1 mRNA (down)	Promotes metastasis in LUAD by activating the Wnt/β-catenin pathway	[260]
	GAS5 mRNA (down)	Promotes metastasis in cervical cancer	[261]
	FGF14-AS2 lncRNA (down)	Promotes osteolytic metastasis in breast cancer	[262]
	circ_SFMBT2 (down)	Promotes metastasis in NSCLC	[263]
	IL-11, SERPINE2 mRNAs (down)	Represses metastasis in HCC	[264]
YTHDF3 (r)	ST6GALNAC5, GJA1, EGFR, VEGFA mRNAs (up)	Promotes brain metastasis and angiogenesis in breast cancer	[53]
	ZEB1 mRNA (up)	Promotes metastasis in HCC and TNBC	[265, 266]
	DICER1-AS1 lncRNA (down)	Promotes pancreatic cancer progression	[267]
	LOXL3 mRNA (up)	Promotes metastasis in melanoma	[268]
	PFKL mRNA (up)	Promotes metastasis in HCC	[269]
FTO (e)	miR‐181b‐3p (down)	Promotes metastasis in breast cancer by upregulating ARL5B	[270]
	TEAD2 mRNA (down)	Represses ICC progression	[271]
FTO (e) YTHDF1 (r)	KRT7 mRNA (up)	Promotes lung metastasis in breast cancer, induction of EMT	[221]
FTO (e) YTHDF2 (r)	BNIP3 mRNA (down)	Promotes metastasis in breast cancer	[272]
	HOXB13 mRNA (up)	Promotes metastasis in endometrial cancer by activating Wnt signaling pathway	[273]
	HSF1 mRNA (up)	Promotes metastasis in multiple myeloma	[274]
ALKBH5 (e)	YAP mRNA (down)	Represses growth and metastasis in NSCLC	[275]
	KCNK15-AS1 lncRNA (up)	Inhibits mobility and invasion of pancreatic cancer cell	[276, 277]
	TGFβR2, SMAD3 mRNA (down), SMAD6 mRNA (up)	Represses metastasis in NSCLC	[278]
ALKBH5 (e) IGF2BP1 (r)	NANOG, KLF4 mRNA (up)	Promotes stem cell phenotype in breast cancer	[279, 280]
ALKBH5 (e) YTHDF2 (r)	KCNQ1OT1 lncRNA (up)	Promotes LSCC tumorigenesis and metastasis via upregulation of HOXA9	[281]
	UBE2T mRNA (up)	Promotes metastasis in HCC lncRNA CASC11- ALKBH5	[282]
	circCPSF6 (up)	Promotes metastasis in HCC by activating YAP signaling	[283]
ALKBH5 (e) YTHDF3 (r)	circ3823 (down)	Represses metastasis in CRC	[284]
m^5^C			
NSUN2 (w)	PIK3R1, PCYT1A mRNAs (up)	Promotes metastasis in gastric cancer	[285]
NSUN2 (w) YBX1 (r)	HDGF mRNA (up)	Promotes metastasis in bladder urothelial carcinoma	[286]
	NKILA lncRNA (up)	Promote growth and metastasis in cholangiocarcinoma	[287]
	LRRC8A lncRNA (up)	Promotes metastasis in cervical cancer	[288]
NSUN3 (w)	mtRNAs of subunits of the oxidative phosphorylation complex	Promotes metastasis in HNSCC	[289]
NSUN7 (w)	CCDC9B mRNA (up)	Prevents liver cancer. Epigenetic loss is associated with worse clinical outcome in HCC	[290]
m^1^A			
ALKBH1 (e)	METTL3 mRNA (up)	Promotes metastasis in CRC by METTL3-mediated downregulation of SMAD7	[291]
ac^4^C			
NAT10 (w)	COL5A1 mRNA (up)	Promotes metastasis in gastric cancer	[292]
	FSP1, KIF23 mRNA (up)	Promotes metastasis in CRC	[293, 294]
	HSP90AA1 mRNA (up)	Promotes ER stress-mediated metastasis in HCC	[295]
	CTC-490G23.2 lncRNA (up)	Promotes metastasis in ESCC	[296]
m^7^G			
METTL1/WDR4 (w)	tRNAs	Promotes metastasis in nasopharyngeal carcinoma through Wnt/β-catenin pathway	[297]
	tRNAs	Promotes metastasis in HCC by enhancing the translation of SNAIL and SLUG	[298]
	tRNAs	Promotes progression of HNSCC through regulating global mRNA translation, including the PI3K/AKT/mTOR signaling pathway	[299]
	miR-760 (up)	Promotes metastasis in bladder cancer by indirectly degrading the tumor suppressor ATF3 mRNA via miR-760	[300]
WBSCR22/ TRMT112 (w)	ISG15 mRNA (down)	Represses tumorigenesis in pancreatic cancer	[301]
mcm^5^s^2^U			
ELP3 (w) CTU1/2	tRNAs	Promote metastasis in breast cancer by enhancing translation of DEK oncoprotein	[302]
Ψ			
Dyskerin (w)	HIF-1α promoter (up)^c^	Promotes metastasis in CRC by directly bound to the HIF-1α promoter to enhance its transcription	[303]
Nm			
FBL (w) SNORD89	BIM mRNA (down)	Involved in endometrial carcinoma lymph node metastasis	[304]
A-to-I editing			
ADAR1 (w)	ITGA2 mRNA (up)	Promotes metastasis in HCC	[305]
	Not determined	Promotes metastasis in gastric cancer via mTOR/p70S6K/RPS6 ribosomal protein signaling axis	[306]
	AZIN1 mRNA (editing)	Promotes metastasis in colorectal and gastric cancer	[307, 308]
	KPC1 mRNA (editing)	Promotes distant metastasis in ICC	[309]
	GLI1 mRNA (editing)	Promotes metastasis in gastric cancer	[310]
	miR-455-5p, miR-378a-3p (editing)	Prevents metastasis in melanoma	[311, 312]
	ITGB3 mRNA (down)^d^	Represses invasion and metastasis in melanoma	[313]
	GABRA3 mRNA (editing)	Represses metastasis in breast cancer	[314]
	circRBMS3 (down)	Represses metastasis in osteosarcoma by sponging miR-424-5p and protecting YRDC/eIF4B	[315]
ADAR2 (w)	miR-200s secretion	Represses lung metastasis in CRC. ADAR2-PKCζ	[316]
	SLC22A3 mRNA (down)	Promotes metastasis in esophageal cancer	[317]
	circRNA-51217 (down)	Represses metastasis in prostate cancer	[318]
	circFNTA (down)	Represses bladder cancer invasion. ADAR2-AR	[319]
C-to-U editing			
APOBEC3G (w)	miR-29 (down)^d^	Promotes hepatic metastasis in CRC through inhibition of miR-29-mediated suppression of MMP2	[320]
	KLF4 mRNA (down)^d^	Promotes oncogenic transformation of cancer cells	[321]

The RNA modifications shown are m^6^A, N6-methyladenosine; m^1^A, N1-methyladenosine; m^5^C, 5-methylcytosine; ac^4^C, N4-acetylcytidine; m^7^G, N7-methylguanosine; mcm^5^s^2^U, 5-methoxycarbonylmethyl-2-thiouridine; Ψ, pseudouridine; Nm, 2′-O-methylation; A-to-I, adenosine-to-Inosine editing, and C-to-U, cytidine-to-uridine editing

Abbreviations: *circ* circular RNA; *CRC* colorectal cancer; *EMT* epithelial to mesenchymal transition; *ESCC* esophageal squamous cell carcinoma; *HCC* hepatocellular carcinoma; *HNSCC* head and neck squamous cell carcinoma; *ICC* intrahepatic cholangiocarcinoma; *lncRNA* long non-coding RNA; *LSCC* laryngeal squamous cell carcinoma; *LUAD* lung adenocarcinoma; *miRNA* microRNA; *mRNA* messenger RNA; *NSCLC* non-small cell lung cancer; *PTC* papillary thyroid cancer; *RCC* renal cell carcinoma; *TNBC* triple-negative breast cancer

^a^ w, writer; r, reader; e, eraser

^b^up, upregulation; down, downregulation

^c^Unrelated with RNA-modifier function of Dyskerin

^d^Unrelated with the editing function of ADAR1 or APOBEC3G

The most known epitranscriptomic modification is certainly the N6-methyladenosine (m^6^A) deposition on mRNAs. The catalytic activity of m^6^A deposition is driven by METTL3 and assisted by METTL14, WTAP, RBM15, KIAA1429, and ZC3H13. The three described *erasers* are FTO, ALKBH3, and ALKBH5, and the known *readers* that bind to the m^6^A mark and mediate cellular outcomes are YTHDF1/2/3, YTHDC1/2, IGF2BP1/2/3, and HNRNPC/A2B1.

Starting with the m^6^A writer, METTL3 has been found upregulated in late stages of breast cancer and associated with worse prognosis [228]. Mechanistically, the authors have shown that METTL3-mediated m^6^A modification of SOX2, one of the regulators of Nanog, promotes stemness and malignant progression of breast cancer [228]. This example highlights that epitranscriptomic regulations of transcription factors (like SOX2) can control EMT effectors (like Nanog) potentially regulated by epigenetic events. In CRC, METTL3 also stabilizes SOX2 mRNA, and promotes drug resistance and lung metastasis [229]. Another METTL3 role in CRC is by downregulating SPRED2 and targeting the miR-1246/SPRED2/MAPK signaling pathway [219]. Moreover, Yin et al. have found that m^6^A methylation can regulate cancer growth and metastasis via macrophage reprogramming [220]. Strikingly, ablation of METTL3 in myeloid cells promoted tumor growth and metastasis, associated with decreased YTHDF1-mediated translation of SPRED2 and increased activation of NF-kB and STAT3 through the ERK pathway, leading to increased tumor growth and metastasis [220]. In a breast cancer lung metastasis model, upregulated METTL3 methylates KRT7-AS to enhance its expression, which then forms duplex with KRT7 mRNA to increase its stability and expression through recruitment of the IGF2BP1/HuR complex [221]. Moreover, YTHDF1/eEF-1 is involved in FTO-regulated translation elongation of KRT7 mRNA [221]. Of note, KRT7 has been described to promote EMT in ovarian cancer via the TGFβ/Smad2/3 signaling pathway [322]. Interestingly, it has been reported in breast cancer that *BRCA1* promoter methylation is correlated strongly and specifically with both *BRCA1* gene expression in ER − tumors and *KRT7* promoter methylation with *KRT7* gene expression in ER + tumors [323]. The same authors also reported differential KRT7 protein expression in luminal cell populations associates with survival [324]. This example underlines that some epigenetically regulated genes can co-participate in tumorigenesis in a way like BRCA1.

Back to METTL3, its oncogenic activity has also been linked to tumor formation in CRC where m^6^A methylation of lncRP11 increases its nuclear accumulation, drives Siah1 and Fbxo45 mRNA degradation, and prevents ZEB1 mRNA decay, thus, regulating EMT and liver metastasis [222]. In HCC, METTL3 decreases SOCS2 mRNA stability (through an m^6^A-YTHDF2-dependent mechanism) and promotes lung metastasis [232]. Interestingly, epigenetic downregulation of SOCS2 has been described in AML cells carrying NRAS mutation, driven by a reduction of the active enhancer marker H3K27ac at the SOCS2 locus [325]. In gastric cancer, METTL3 stabilizes hepatoma-derived growth factor (HDGF) that activates GLUT4 and ENO2 expression and subsequent angiogenesis and glycolysis that promote liver metastasis [223]. Of note, concomitant upregulation of HDGF and PRKCA has been associated with poor prognosis in lung adenocarcinoma [326]. Conversely, in a study in papillary thyroid cancer (PTC), low level of STEAP2 correlated with aggressiveness, and METTL3-mediated STEAP2 mRNA stabilization decreased PTC progression [224]. In cervical cancer, METTL3 is upregulated, promoting tumorigenesis and Warburg effect, and correlates with lymph node metastasis and poor prognosis [230]. Mechanistically, METTL3 targets the 3′UTR of the hexokinase HK2 mRNA, and recruits YTHDF1 to enhance its stability [230]. In hepatoblastoma, a rare liver cancer usually diagnosed during the first 3 years of life, the upregulation of METTL3, YTHDF2, and FTO has been correlated with poor clinical outcomes. METTL3 knockdown in hepatoblastoma cells could suppress proliferation, invasion, and migration [327]. In melanoma, METTL3-mediated stabilization of the uridine cytidine kinase 2 (UCK2) by m^6^A modification plays a role in melanoma metastasis by enhancing the Wnt/β-catenin pathway [225]. Of note, LINC00470 recruits METTL3 to drive m^6^A methylation of PTEN mRNA, leading to YTHDF2-dependent PTEN mRNA decay and finally promoting metastasis in gastric cancer [233]. In NSCLC, Pan et al. found that the METTL3/YTHDF2 m^6^A axis also downregulates miR-1915-3p that has tumor suppressor function by targeting SET nuclear proto-oncogene (SET) [234]. In a more functional study, Li et al. suggested that METTL3 regulates EMT by modulating β-catenin expression and subcellular localization [328]. Moreover, by studying TGFβ-mediated EMT in cancer cell lines, a significant increase of m^6^A levels in cells undergoing EMT has been described. Particularly, m^6^A deposition in SNAI1 mRNA triggers its YTHDF1-mediated translation to promote EMT [231]. Interestingly, the microbiota can also exert a strong influence on the host m^6^A epitranscriptome. A recent study has shown that gut microbes *Fusobacterium nucleatum* reduce the m^6^A levels of CRC cells through the YAP/FOXD3/METTL3 axis, resulting in upregulation of KIF26B, enhanced aggressiveness, and metastasis [329]. From a therapeutic standpoint, the screening of a library of small molecules identified Elvitegravir, originally developed to treat human immunodeficiency virus (HIV) infection, as a drug able to repress the invasion and metastasis of ESCC cells by enhancing the proteasomal degradation of METTL3 mediated by STUB1. The authors have shown that METTL3-mediated m^6^A modified EGR1 mRNA is stabilized by YTHDF3, leading to increased activation of EGR1/Snail signaling [241]. In breast cancer cells, METTL3 mediates methylation of the lncRNA MALAT1 that promotes metastasis. MALAT1 acts as a decoy for miR-26b, which targets and downregulates HMGA2. Thus, m^6^A methylation of MALAT1 indirectly increases HMGA2 expression, with an effect on EMT. Notably, high expression of METTL3 and MALAT1 in breast cancer has been associated with poor prognosis [226]. In lung cancer, Wang et al. have shown that METTL3 targets miR-143-3p to enhance its biogenesis, leading to the silencing of vasohibin-1 (VASH1) and subsequent increased angiogenesis and blood–brain barrier invasion capability, finally promoting brain metastasis [227]. Conversely, METTL14 acts as a metastasis suppressor by mediating the decay of its targets via recognition by m^6^A readers. Examples include m^6^A-dependent degradation of NEAT1 lncRNA [242] and ITGB4 mRNA [243] in RCC; or SOX4 mRNA [244] and XIST lncRNA [245] in CRC, all of them via recognition by YTHDF2. Moreover, METTL14 was found downregulated in CRC, and decreased expression correlates with poor overall survival [244].

Regarding m^6^A readers, YTHDF1 expression correlates with lymph node metastasis and worse clinical stages in CRC. The oncogene c-MYC promotes the YTHDF1 expression in CRC cells [330]. YTHDF1 has been directly implicated in EMT by controlling Snail mRNA modification and translation rate [231]. YTHDF1 promoted tumorigenesis and metastasis in ovarian cancer by augmenting the translation of m^6^A-modified eIF3C mRNAs [246]. eIF3C upregulation has been associated with tumorigenesis in RCC [331] and HCC, in the later by increasing the secretion of extracellular exosomes to promote angiogenesis [332]. Moreover, simultaneous high expression of EIF3C and S100A11 is associated with poor HCC patient survival [332]. In HCC, a clinically relevant m^6^A-YTHDF1-EGFR axis has been described. Radiofrequency ablation (RFA) is recommended as a minimally invasive curative therapy in this tumor type. However, a recurrent rate of 50% is observed within 3 years, mainly attributed to metastasis due to sublethal heat stress from insufficient RFA. A recent study has shown that out of the ablation center, sublethal heat treatment in the surrounding transitional zone results in cellular stress that promotes YTHDF1 expression and could contribute to HCC metastasis. Mechanistically, YTHDF1-mediated recognition of m^6^A-modified EGFR mRNA enhanced its translation, supporting the rationale for targeting m6A machinery in combination with EGFR inhibitors to prevent metastasis in HCC after RFA [247]. Also in HCC, HIF1α-induced expression of YTHDF1 drives ATG2A and ATG14 translation, two autophagy-related genes, promoting tumor progression [248]. Other examples of the pro-metastatic role of YTHDF1 by promoting gene translation have been described, including YTHDF1-mediated increased translation of USP14 in gastric cancer [249], ARHGEF2 in CRC [250], FOXM1 and PKM2 in breast cancer [251, 252], PLK1 in prostate cancer [253], SLP2 and ANLN in HCC [254, 255], DDX23 in pancreatic ductal adenocarcinoma [256], and HRAS in various cancers [257]. In breast cancer, YTHDF1 and YTHDF3 are frequently amplified and consequently overexpressed, and significant correlations with intrinsic subclasses and nodal metastasis have been described, suggesting their use for prognosis stratification and therapeutic intervention in breast cancer [333]. Regarding regulation, YTHDF1 can be targeted by miRNAs, including miR-136-5p in CRC [334] and also miR-1285-3p in lung cancer, where Linc00337 is upregulated and acts an miR-1285-3p sponge enhancing YTHDF1 expression [335]. The study in lung cancer also has shown that Linc00337 silencing represses tumor progression *in vitro* and *in vivo* [335].

About YTHDF2, the METTL3/YTHDF2 m^6^A axis promotes tumorigenesis in bladder cancer by mediating degradation of RRAS [235], SETD7, and KLF4 [236]. Also, METTL3 depletion suppressed metastasis [236]. In HCC, YTHDF2 expression has been correlated with poor survival, and it drives the translation of OCT4 [258]. Moreover, the loss of YTHDF2 led to reduction of tumor burden and lung metastasis in mice [258]. Also in HCC, m^6^A-modified FYN mRNA bound YTHDF2 and the proliferation associated protein 2G4 (PA2G4). Thus, PA2G4 upregulation in HCC promotes EMT, and plays a pro-metastatic role by increasing FYN expression through binding with YTHDF2 [259]. In lung adenocarcinoma, YTHDF2 is upregulated and drives AXIN1 mRNA decay, activating the Wnt/β-catenin signaling pathway that finally promotes tumorigenesis [260]. In cervical cancer, YTHDF2-mediated degradation of the tumor suppressor GAS5 enhances growth and metastasis, and inversely, the lncRNA GAS5-AS1 has the opposite effects [261]. In breast cancer, YTHDF2 downregulates FGF14-AS2, promoting osteolytic metastasis by enhancing RUNX2 mRNA translation [262], and in NSCLC, it downregulates circ_SFMBT2 to promote metastasis [263]. Other examples supporting the role of the METTL3/YTHDF2 m^6^A axis in metastasis include the decay of targets with tumor suppressor functions, as YPEL5 (associated with decrease of CCNB1 and PCNA expression) in CRC [237], USP4 in prostate cancer [238], DUSP5 in gallbladder cancer [239], and IFFO1 in different types of cancer [240]. Nevertheless, there are also examples associating a decrease in YTHDF2 expression with poor prognosis, tumor progression, and metastasis in HCC [264]. YTHDF2 degrades m^6^A-containing IL-11 and SERPINE2 mRNAs, two mediators of hypoxia-induced cancer cell survival and vascular reconstruction. Thus, HIF-2α-mediated inhibition of YTHDF2 in HCC promotes cancer-associated inflammation. Importantly, the use of HIF-2α antagonist restored YTHDF2-programed epigenetic machinery and repressed liver cancer [264]. Also, in lung adenocarcinoma, YTHDF2 was found to inhibit migration and invasion, and high expression correlated with better overall survival [336].

The third member of the YTH family, YTHDF3, has also been implicated in metastasis. In HCC, ZEB1 mRNA is upregulated by circ_KIAA1429 in a YTHDF3 m^6^A-dependent manner, leading to enhanced invasion and metastasis [265]. Moreover, in TNBC, increased expression of YTHDF3 correlated with worse prognosis, and YTHDF3 positively regulated cell migration, invasion, and EMT by also stabilizing ZEB1 mRNA [266]. As previously commented, YHTDF1 and YTHDF3 are amplified and overexpressed in breast cancer, correlating with poor overall survival and nodal metastasis [333]. In breast cancer brain metastasis, YTHDF3 overexpression due to increased copy number has been also described [53]. YTHDF3 induces the translation of ST6GALNAC5, GJA1, EGFR, and VEGFA mRNAs in an m^6^A-dependent manner [53]. In pancreatic cancer, YTHDF3 increased expression correlates with poor overall survival, and YTHDF3 regulates the lncRNA DICER1-AS1 that promotes glycolysis through inhibition of miR-5586-5p [267]. The pro-metastatic effect of YTHDF3 was also observed in melanoma, where it increases the translation of LOXL3 mRNA [268]. In HCC, YTHDF3 expression was found upregulated and related to worse prognosis, and it promotes aerobic glycolysis by increasing the translation of PFKL [269].

About m^6^A erasers, the FTO demethylase is upregulated in breast cancer and associated with poor overall survival [272]. The study demonstrated that FTO targets m^6^A in the 3′UTR region of the pro-apoptotic BNIP3 mRNA, suggesting FTO as a potential therapeutic target to enhance BNIP3 expression and decrease tumor growth and metastasis [272]. In RCC, BNIP3 is epigenetically silenced by histone deacetylation [337], and treatment with the HDAC inhibitor (HDACi) trichostatin A restores BNIP3 expression and led to cell growth inhibition and apoptosis [337], suggesting a potential therapeutic strategy in that setting. This strategy was also proposed in TNBC, where the combination of the HDACi YCW1 with radiation induced autophagic cell death by downregulation of BNIP3 [338]. This combination has also been proposed to tackle breast cancer lung metastasis [339]. Also in breast cancer, FTO upregulates ARL5B by inhibiting miR‐181b‐3p [270]. In endometrial cancer, FTO demethylates HOXB13 mRNA, promoting metastasis by activating Wnt signaling pathway [273]. In this cancer type, FTO was upregulated in metastases, and demethylation of HOXB13 mRNA led to a decreased decay by lack of YTHDF2 recognition [273]. In multiple myeloma (MM), FTO has also a pro-metastatic role by targeting HSF1, enhancing HSF1 mRNA stability and translation [274]. Importantly, FTO inhibition combined with bortezomib treatment synergistically inhibited myeloma bone tumor formation [274]. Nevertheless, a tumor suppressor role of FTO has been described in intrahepatic cholangiocarcinoma (ICC) by controlling different pathways, including EGFR, and by decreasing the stability of the oncogene TEAD2 [271].

Regarding the ALKBH5 m^6^A eraser, exposure to hypoxia induced the HIF-1α and HIF-2α-dependent expression of ALKBH5 that demethylates and stabilizes Nanog mRNA, leading to the acquisition of a cancer stem cell phenotype in breast cancer cells [279]. Moreover, inhibitions of either ALKBH5 or HIF factors were effective strategies to decrease Nanog expression. The same authors observed that hypoxia also leads to the overexpression of Nanog and another pluripotency factor, KLF4, in a ZNF217-dependent and HIF-dependent manner in breast cancer cells [280]. Interestingly, ALKBH5 knockdown in a breast cancer cell line decreased metastasis from breast to lung *in vivo* [280]. In laryngeal squamous cell carcinoma (LSCC), increased expression of ALKBH5 can promote tumorigenesis in an m^6^A‐YTHDF2‐dependent manner by promoting the lncRNA KCNQ1OT1‐HOXA9 signaling axis [281]. In HCC, the lncRNA CASC11 is upregulated and associated with poor survival and metastasis [282]. CASC11 stabilizes UBE2T mRNA by recruiting ALKBH5 m^6^A activity and also by inhibiting UBE2T mRNA association with YTHDF2 [282]. Also in HCC, YAP activation via the ALKBH5-mediated m^6^A demethylation of circCPSF6 has been associated with malignancy, as circCPSF6 sustains the stability of YAP1 mRNA [283]. m^6^A-modified circCPSF6 is recognized and destabilized by YTHDF2, but circCPSF6 m^6^A demethylation by ALKBH5 facilitates the proliferation and metastasis of HCC cells via competitive interaction of circCPSF6 with PCBP2, activating the YAP1 signaling [283]. In CRC, another circRNA involved in progression and metastasis is circ3823. The authors have shown that circ3823 could be targeted by YTHDF3 and ALKBH5 to promote its degradation; meanwhile, these modifiers are downregulated in CRC, leading to increased expression of circ3823. Circ3823 repressed miR-30c-5p that normally impedes transcription factor 7 (TCF7) mRNA translation, leading to increased TCF7 protein level and promoting proliferation, angiogenesis, and metastasis [284]. However, in NSCLC, ALKBH5 inhibited growth and metastasis by reducing YTHDF-mediated YAP expression [275]. In pancreatic cancer cells, ALKBH5 also inhibits cell motility and invasion by demethylation-dependent increase of KCNK15-AS1 expression [276, 277]. Mechanistically, KCNK15-AS1 is able to recruit MDM2 to promote REST ubiquitination, thus transcriptionally upregulating PTEN to inactivate AKT pathway [277]. In NSCLC, ALKBH5 overexpression decreases the mRNA stability and expression of TGFβR2 and SMAD3 but enhances those of SMAD6, leading to a reduction of metastasis *in vivo* [278]. About the ALKBH3 eraser, one study linked its overexpression to metastasis and poor progression-free and overall survival in HCC [340].

Another epitranscriptomic modification that has been implicated in metastasis is 5-methylcytosine (m^5^C). This modification is written by the NOP2/Sun RNA methyltransferase family members NSUN1 to NSUN7, the DNA methyltransferase DNMT2, or the TRNA aspartic acid methyltransferase 1 TRDMT1, depending on the RNA target. Thus, for example, NSUN1 and NSUN5 modify rRNAs; NSUN2, NSUN6, and DNMT2 methylate tRNAs; NSUN3 and NSUN4 modify mitochondrial RNAs (mtRNAs), whereas NSUN2, NSUN6, and DNMT2 methylate mRNAs [341–343]. In addition, our group has recently shown that NSUN7 is also able to deposit the m^5^C mark in mRNA, characterizing the transcript of coiled-coil domain containing 9B (*CCDC9B*) gene as a relevant target in HCC. Interestingly, we have shown that the epigenetic-mediated inactivation of *NSUN7* in liver cancer decreases CCDC9B mRNA half-life and is associated with poor overall survival [290]. Few years ago, we also described the epigenetic silencing of *NSUN5* by gene-promoter methylation in glioma. NSUN5 methylates cytosine at position C3782 of 28S rRNA and its loss drives an adaptive translational program. In contrast to NSUN7, NSUN5 silencing is a hallmark of glioma patients with long-term survival [344]. These examples further support the crosstalk between epigenomic and epitranscriptomic mechanisms as a key player in cancer. Regarding m^5^C readers, the first identified was the Aly/REF export factor (ALYREF) [345]. Also, Y-box binding protein 1 (YBX1) recognizes and enhances the stability of m^5^C-modified mRNA by recruiting ELAVL1 [286].

In breast cancer, NSUN2 is overexpressed by gene promoter hypomethylation and correlates with clinical stages [346]. In gastric cancer, NSUN2 overexpression has been shown associated with metastasis and regulated by SUMO-2/3 that stabilized and mediated its transport into the nucleus. PIK3R1 and PCYT1A expression correlated with prognosis and NSUN2 expression, and they could be the target genes that participate in gastric cancer progression [285]. In bladder cancer, NSUN2 methylates and stabilizes HDGF mRNA 3′UTR (also stabilized by METTL3 in gastric cancer) promoting cancer metastasis [286]. Moreover, a high co-expression of NUSN2, YBX1, and HDGF predicts poor survival in this tumor type [286]. NSUN2 overexpression and NSUN6 downregulation were associated with poor prognosis in TNBC by upregulating monocytes/macrophages and Tregs in the tumor immune microenvironment [347]. Recently in cholangiocarcinoma, Zheng et al. have shown that NSUN2 targets the NF-kB interacting lncRNA NKILA enabling the recognition by YBX1. They also showed that NKILA targets YAP1 via miR-582-3p and the upregulation of NKILA was correlated with lymph node and distant metastasis [287]. LRRC8A is another lncRNA that has been demonstrated targeted by NSUN2 and stabilized by YBX1 in cervical cancer. In this context, NSUN2 expression was associated with metastasis [288]. Regarding NSUN3, considering its function as mtRNA modifier, and the metabolic flexibility and plasticity during cancer progression [348], NSUN3 could play a role in the metabolic adaptability required for metastasis formation. Indeed, a pivotal study in the field has recently shown that m^5^C in mtRNAs is essential for the dynamic regulation of mitochondrial translation rates, and thereby shapes metabolic reprogramming during metastasis [289]. The authors have shown that the translation of mitochondrially encoded subunits of the oxidative phosphorylation complex depends on the formation of m^5^C at position 34 in mitochondrial tRNA^Met^. Importantly, this modification was required for efficient tumor metastasis, but not for primary tumor development and growth [289]. This study also identified an NSUN3-driven gene signature that predicts metastasis in patients with HNSCC. Remarkably, these findings support the inhibition of m^5^C formation in mitochondria as a therapeutic opportunity to prevent the dissemination of tumor cells from primary tumors [289]. Also in HNSCC, NSUN5 and ALYREF expression correlated with TNM stages [349], underlying again the different routes cancer cells can exploit to increase the metastatic competence. Moreover, the tRNA m^5^C methyltransferase TRDMT1 has been positively associated with tumor size, histological grade, invasion depth, lymph node metastasis, and TNM stage in gastric cancer [350].

Regarding other RNA modifiers, TRIT1 (tRNA isopentenyltransferase 1) is the enzyme responsible for the hypermodification of the A37 position in the anticodon region of human tRNAs containing serine and selenocysteine to N6-isopentenyladenosine (i^6^A). A TRIT1 polymorphism and a haplotype were associated with lymph node metastasis in gastric cancer [351]. Moreover, our group has found that TRIT1 undergoes gene amplification-associated overexpression in small cell lung cancer (SCLC) that promotes tumor growth [352]. SCLC is a disease characterized by aggressive dissemination and poor prognosis. In most of the cases, the cancer has already metastasized by the time of diagnosis. Another modification affecting adenine is N1-methyladenosine (m^1^A). Like other modifications, its deposition is dynamically regulated by several proteins: the tRNA methyltransferases TRMT61A, TRMT61B, and TRMT10C assisted by TRMT6 that introduce m^1^A, and the demethylases ALKBH1 and ALKBH3 that remove the modification. Moreover, YTHDF1, YTHDF2, YTHDF3, and YTHDC1 recognize m^1^A sites and induce downstream effects. In CRC, it has been recently shown that MFAP2 mRNA was overexpressed and highly m^1^A modified, and its expression was related to lymph node metastasis and distant metastasis, leading to poor prognosis. Nevertheless, the authors did not elucidate the TRMT writer implicated in this process [353]. TRMT6 non-catalytic subunit is overexpressed and associated with the TNM stage in HCC, through the PI3K/AKT/mTOR axis, and it has been suggested as potential therapeutic target for this tumor type [354]. Moreover, a recent study has found that the demethylase ALKBH1 is overexpressed in CRC and associated with metastasis and poor prognosis. The study showed that ALKBH1-catalyzed m^1^A demethylation is essential for CRC cell migration and invasion, suggesting that ALKBH1 accelerates CRC metastasis by downregulating SMAD7 expression. Mechanistically, the authors described that ALKBH1-mediated m^1^A demethylation of METTL3 mRNA inhibits SMAD7 expression by METTL3-mediated m^6^A modification [291]. YTHDC2 is an m^1^A reader that has been related to regulation of EMT effectors and metastatic ability of breast cancer cells [355].

NAT10, the writer of N4-acetylcytidine (ac^4^C), is another epitranscriptomic player that has been implicated in various types of cancer. As previously commented, a role of NAT10 as metastasis suppressor has been found in CRC, where miR-6716-5p-dependent downregulation of NAT10 enhances metastasis [356]. Nevertheless, several studies have described an opposite role. For instance, in gastric cancer, NAT10 promotes metastasis and EMT by targeting COL5A1 mRNA and increasing its stability [292]. Also, NAT10 is upregulated in colon cancer and associated with poor overall survival. In this setting, NAT10 inhibits ferroptosis through N4-acetylation and stabilization of the ferroptosis suppressor protein 1 (FSP1) mRNA [293]. Additional roles of NAT10 include ac^4^C modification of targets as the HSP90AA1 mRNA in ER stress-mediated metastasis and lenvatinib resistance in HCC [295], the CTC-490G23.2 lncRNA that interacts with PTBP1 to increase CD44 alternative splicing in primary ESCC [296], and the KIF23 mRNA to regulate the Wnt/β-catenin signaling pathway in colorectal cancer [294].

N7-methylguanosine (m^7^G) is another epitranscriptomic modification with critical roles in the regulation of gene expression that is dysregulated in several diseases, including cancer. m^7^G was originally identified in mRNAs, but later found at defined internal positions within multiple types of RNAs, including tRNAs, rRNAs, and miRNAs [357]. The most common m^7^G writer is METTL1, in complex with the non-catalytic subunit WDR4. In nasopharyngeal carcinoma (NPC), METTL1/WDR4 promotes tumorigenesis via regulation of Wnt/β-catenin, and their upregulation has been associated with poor prognosis [297]. Moreover, as previously commented for YTHDF1 [247], the same team found that insufficient radiofrequency ablation of HCC also results in a significant upregulation of METTL1/WDR4 and m^7^G levels in tRNAs that could promote metastasis by enhancing the translation of SLUG/SNAIL EMT regulators [298]. In addition, METTL1/WDR4-mediated m^7^G modification of tRNAs could promotes tumor progression and metastasis by enhancing the translation of a subset of oncogenic transcripts in HNSCC, including genes related to the PI3K/AKT/mTOR signaling pathway [299]; or by promoting the processing of miR-760 in an m^7^G modification-dependent way and indirectly degrading the tumor suppressor ATF3 mRNA via miR-760 in bladder cancer [300]. Besides, m^7^G modifications in 18S rRNA are introduced by the WBSCR22/TRMT112 methyltransferase complex. Decreased expression of WBSCR22 has been observed in inflammatory and neoplastic lung pathologies [358]. WBSCR22 enhances glucocorticoid receptor (GR) function through binding to the GR co-activator GRIP1. Its expression is decreased by cytokines TNFα and IFNγ by driving ubiquitination of two conserved lysine residues [358]. In pancreatic cancer, WBSCR22/TRMT112 negatively regulates the transcription of ISG15, diminishing proliferation, migration, invasion, and globally tumorigenesis [301]. Nevertheless, WBSCR22 overexpression has been described in glioma and correlates with poor prognosis [359].

Another methylation event that provides tRNA stability and enhance translation occurs at position 10 in tRNA, the N2-methylguanosine (m^2^G), introduced by TRMT11. Interestingly, TRMT11 gene fusion with GRIK2 (TRMT11-GRIK2) has been identified as a frequent event in several tumor types, including primary and lymph node metastatic cancer from breast, colon, and ovary [360]. GRIK2 encodes a glutamate receptor with tumor suppressor features. Chromosomal recombination between TRMT11 and GRIK2 destroys the open-reading frames of both genes and produces functional knockouts of these two proteins. Consequently, lack of TRMT11 may produce less efficient and unstable translation due to tRNA defects, or protein translation repression [360]; plus the loss of GRIK2 tumor suppressor activity. Further investigations are guaranteed to unravel the functional consequences of this genetic lesion.

Regarding modifications in uridine, the wobble uridine of specific tRNAs is transformed to 5-methoxycarbonylmethyl-2-thiouridine (mcm^5^s^2^U), which is critical for proper mRNA decoding and protein translation. ELP3 is the enzymatic core of the elongator complex that promotes this modification [361]. DNA hypermethylation-mediated decrease of ELP3 has been detected in invasive ductal breast cancer [362]. In contrast, examples of pro-metastatic roles of ELP3 have also been described. ELP3 expression has been correlated with lymph node metastasis in endometrioid adenocarcinoma [363]. In breast cancer, ELP3 together with its partners CTU1/2 are upregulated and sustain metastasis by driving the translation of the oncoprotein DEK in a mcm^5^s^2^-tRNA-dependent manner. Furthermore, DEK promotes the IRES-dependent translation of the pro-invasive transcription factor LEF1 [302]. Of note, DEK has been shown regulated by gene promoter hypomethylation in HCC [364]. Also, CTU1 was one of the nine genes used to generate a risk score model in prostate cancer that recognize patients with worse prognosis and higher proportions of lymph node metastasis [365]. CTU1 has also been included in two analogous risk score models of bladder cancer [366, 367].

Uridines are also target of pseudouridylation. Pseudouridine (Ψ) is the most abundant RNA modification and the first to be discovered, more than 70 years ago. *DKC1* gene codes for Dyskerin, the catalytic subunit of H/ACA small nucleolar ribonucleoprotein (H/ACA snoRNP) complex, which catalyzes pseudouridylation of rRNA. Dyskerin also plays a role in telomerase stabilization. DKC1 expression has been related to unfavorable TNM stages in ccRCC [368]. In CRC, Dyskerin facilitates angiogenesis and metastasis via increased expression of HIF-1α and VEGF by a mechanism unrelated to its RNA-modifier role. Dyskerin directly bound to the HIF-1α promoter and enhance its transcription and promote CRC progression [303]. Moreover, a recent meta-analysis of nine studies confirmed the link between Dyskerin overexpression and metastasis [369]. In pancreatic ductal adenocarcinoma, SNORA23, an H/ACA-box type small nucleolar non-coding RNA participating on pseudouridylation, was found upregulated and associated with metastasis formation by increasing the expression of spectrin repeat-containing nuclear envelope 2 (SYNE2) [370]. In addition, in gastric cancer, Liu et al. found that the overexpression of SNORA21 was associated with increased lymph node metastasis and distant metastasis [371]. However, these studies did not determine if SNORA23 pseudouridylation activity was involved in SYNE2 expression or the potential targets of SNORA21. In ovarian cancer, SNORA70E promotes tumorigenesis through pseudouridylation of RAP1B but direct correlation with metastasis was not established [372]. Thus, further studies are needed to uncover a potential deregulation of pseudouridylation in metastasis.

About 2′-O-methylation (Nm, where N stands for any nucleotide), it is an abundant and highly conserved modification found at multiple locations in tRNAs, rRNAs, mRNAs, and small non-coding RNAs [373]. Fibrillarin (FBL) is a 2′-O-methyltransferase that binds to box C/D snoRNAs, like SNORD89, to form a snoRNP complex and targets rRNA, tRNA, and other RNAs. For instance, a recent study has shown that SNORD89 combined with FBL increased the level of 2′-O-methylation of BIM mRNA, affecting the complementary base pairing of BIM mRNA, thus reducing the pro-apoptotic BIM protein [304]. Moreover, SNORD89 is overexpressed in endometrial carcinoma with lymph node metastasis [304]. Also, FBL is highly expressed in HCC and correlates with lung metastasis and poor prognosis [374]. In NSCLC, SNORD88C was observed upregulated and associated with metastasis. The authors have shown that SNORD88C guides 2′-O-methylation of 28S rRNA that increases SCD1 translation and consequently upregulates MUFA expression, leading to autophagy inhibition and promoting growth and metastasis [375].

RNA editing is another epitranscriptomic event that affects RNA molecules. RNA editing changes the RNA sequence, and consequently it can dramatically alter the amino acid composition and properties of the mRNA-derived protein. Recent studies indicate that many thousands of transcripts are affected by RNA editing, including not only mRNAs but also non-coding RNAs. Two families of proteins carry out editing by deamination: the adenosine deaminases acting on RNA (ADARs), which convert adenosine to inosine (A-to-I); and the apolipoprotein B mRNA editing (APOBECs), which convert cytosine to uracil (C-to-U) [376].

Regarding the A-to-I editing, it is catalyzed by ADAR1, ADAR2, and ADAR3. Inosines are read as guanosines during translation, which has important functional implications. Pro-metastatic roles for ADAR1 have been described in several studies. In HCC, ADAR1 upregulates integrin ITGA2 and drives metastasis by enhancing adhesion of tumor cells to the extracellular matrix [305]. Moreover, aberrant overexpression of ADAR1 in gastric cancer promotes metastasis via mTOR/p70S6K/RPS6 axis activation [306]. In TNBC, ADAR1 high expression and low tumor-infiltrating lymphocytes define worse disease-free survival in patients with lymph node metastasis [377]. In CRC, high ADAR1 expression increases editing of AZIN1. Importantly, elevated level of AZIN1 editing has been identified as an independent risk factor for lymph node and distant metastasis, and as a prognostic factor for overall survival and disease-free survival in CRC patients [307], later confirmed in gastric cancer by the same group [308]. Also, it has been shown that CPEB3 interacts with the 3′UTR of ADAR1 mRNA and inhibits its translation by localizing it to processing bodies (P bodies). CPEB3 is downregulated in gastric cancer. Remarkably, CPEB3 re-expression inhibited gastric cancer growth and metastasis by decreasing ADAR1 expression, opening a therapeutic opportunity [378]. In intrahepatic cholangiocarcinoma, ADAR1 overexpression and concomitant KPC1 overediting lead to distant metastasis [309]. ADAR1 has also been linked to circular RNAs. In gastric cancer, hsa_circ_0004872 circRNA was downregulated by ADAR1 and associated with tumor size and local lymph node metastasis [379]. Next, the circNEIL3, upregulated in PDAC, enhances ADAR1-mediated editing of GLI1 by sponging the miR-432-5p that targets ADAR1 [310]. This mechanism promoted cell cycle progression and EMT. Moreover, high circNEIL3 levels were associated with poor prognosis or TNM stage [310]. ADAR1 has also been suggested as a therapeutic target against gastric cancer metastasis, since ADAR1 knockdown suppressed peritoneal metastasis of gastric cancer in mouse models partially by inhibiting Wnt/β-catenin pathway and EMT [380]. Very recently, Ding et al. designed a nanovesicle as RNA interference tool to overcome resistance to immune checkpoint blockade therapies [381]. The vesicle with siADAR1 silenced tumor cell ADAR1 expression and increased type I/II interferon production, making cells more sensitive to secreted effector cytokines such as IFN-γ with significant cell growth arrest [381].

Although most of the studies have described pro-tumorigenic and metastatic roles for ADAR1, a low expression of ADAR1 has been observed in melanoma metastasis. In this setting, ADAR1-mediated editing of miR-455-5p inhibits metastasis, while unedited miR-455-5p form promotes melanoma metastasis through inhibition of the CPEB1 tumor suppressor gene [311]. Also in melanoma, ADAR1-mediated editing of miR-378a-3p prevents melanoma progression by targeting the PARVA oncogene [312]. Moreover, the same group described a role of ADAR1 in melanoma cell invasion by controlling ITGB3 expression independently of RNA editing [313], regulated by miR-30a and miR-30d [382]. In breast cancer, it was shown that GABAA receptor alpha3 (GABRA3), normally expressed in brain, is upregulated and drives cancer progression and metastasis. Interestingly, the A-to-I edited form of GABRA3 had opposite properties [314]. ADAR1 could also downregulate circRBMS3, a circRNA that promotes osteosarcoma progression by sponging miR-424-5p and protecting YRDC/eIF4B [315]. Thus, ADAR1 downregulation in osteosarcoma could be at least partially responsible for circRBMS3 overexpression, enhancing proliferation and metastasis [315].

Regarding ADAR2, in liver metastasis of CRC, ADAR2 has been identified as a direct substrate of the tumor suppressor protein kinase PKCζ [316]. The authors showed that PKCζ-mediated ADAR2 phosphorylation activates its gene editing activity, which is required to maintain miR-200 steady-state levels. Remarkably, loss of PKCζ in metastasis is associated with lower ADAR2 activity, which promotes the loading of miR-200s into extracellular vesicles, thereby decreasing the intracellular steady-state levels of miR-200s promoting an EMT/stemness phenotype and metastasis [316]. ADAR2 has also been described as driver of invasion and metastasis in esophageal cancer by editing and downregulating the tumor suppressor SLC22A3 [317]. Moreover, the role of androgen receptor (AR) in prostate and bladder cancer invasion linked to ADAR2 has been identified [318, 319]. In prostate cancer, R-2HG accumulation in IDH1 mutated cells increases cell invasion via enhancing TGFβ1/p-Smad2/3 signaling, and AR can reverse this mechanism. AR expression increases ADAR2 expression and negatively regulates circRNA-51217 production and TGFβ1/p-Smad2/3. Restoring AR in cancer cells increases ADAR2 expression and diminishes circRNA generation, which finally resulted in interruption of R-2HG effect [318]. Moreover, this study highlights a therapeutic potential of bipolar androgen therapy (BAT) for patients with IDH1 mutation and low AR expression [318]. In bladder cancer, AR increases the levels of circFNTA to promote cancer cell invasion and cisplatin chemoresistance [319]. In this case, AR strikingly downregulates ADAR2 by binding its 5′ promoter region to increase circFNTA levels, which sponges miR‐370‐3p and increased expression of its host gene FNTA. High level of FNTA activates KRAS to promote cell invasion and chemo‐resistance. Thus, depletion of circFNTA suppressed cancer cell invasion and increased cisplatin chemo‐sensitivity [319].

The C-to-U editing process, driven by APOBEC enzymes, is also relevant in metastasis. APOBEC3G promotes CRC hepatic metastasis through inhibition of miR-29-mediated suppression of MMP2 [320]. Overexpression of APOBEC3G also correlates with worse prognosis and increased risk of hepatic metastasis in patients with CRC [383], and might be a risk factor for HCC progression [384]. Interestingly, it has been shown that APOBEC3G increased expression in cancer cells repressed the expression of the tumor suppressor KLF4 by binding to its mRNA [321]. In parallel, SP1 was seen overexpressed and controlling the increased expression of c-myc, Bmi-1, BCL-2, and MDM2, and decreased of p53, supporting its oncogenic role [321]. Moreover, Zhou and Guo observed that APOBEC3G is increased in CRC metastasis by copy number variation [385]. In melanoma, APOBEC3G was recently identified within a signature of genes modulated by combined inhibition of PLK1 and NOTCH [386], providing a potential therapeutic opportunity. Finally, the Apobec-1 complementation factor (A1CF) promoted progression of RCC through DKK1-MEK/ERK signaling pathway [387], without assessing RNA edition. The same group also described that A1CF promotes RCC cell migration by promoting nuclear translocation of SMAD3 [388]. A1CF overexpression correlated positively with SMAD3, Snail1, and N-cadherin expression [388]. These two studies investigated the role of a co-factor of APOBEC1, suggesting that complementary studies are needed to determine if edition is altered in RCC.

## Final remarks and future perspectives

Metastasis is a threatening progression of cancer, a Damocles sword that hangs over patients. Massive efforts are done to better understand the molecular mechanisms governing tumor progression, aimed to develop better strategies to tackle metastasis, the cause of a great majority of cancer-associated deaths. Metastasis is a dynamic process that requires a rapid phenotypic adaptation to support every stage of the process. As outlined above, the plasticity and reversibility of epigenetic and epitranscriptomic mechanisms make them ideal orchestrators of this dynamic process.

Cancer cells can exploit several strategies to reprogram the expression landscapes to acquire the features that facilitate escape from the primary tumor, migration to distant sites, and invasion of new organs. For instance, cells can exploit epigenetic or epitranscriptomic mechanisms to control core transcription factors such as the EMT regulator ZEB1, modulated by not only miR-200s [94], HDAC1/2 in pancreatic cancer [141, 142], and SIRT1 in prostate cancer [143], but also METTL3 that promotes liver metastasis in CRC [222], or YTHDF3 that promotes metastasis in HCC and TNBC [265, 266]. Why pre-metastatic cells use one or another regulatory mechanism is not fully understood, but we can speculate that intrinsic tumor cell biology, as well as external pressures including microenvironment and treatments, could be major determinants. Moreover, different levers are actioned in specific settings and can have opposite effects depending on the context, like SETDB1 anti-metastatic or pro-metastatic role in breast cancer [135], or in HCC [136], respectively.

As herein reviewed, epigenetic and epitranscriptomic changes play a crucial role in cancer metastasis. Epigenetic alterations regulate key players of the metastatic process (Table 1). For example, promoter-associated DNA methylation silences the expression of several tumor or metastasis suppressor genes, whereas DNA hypomethylation of pro-metastatic genes leads to their upregulation. Together with DNA methylation, histone modifications can modulate gene expression by altering the chromatin accessibility to transcription factors and other regulatory proteins. Non-coding RNAs, such as microRNAs and lncRNAs, can regulate the expression of metastasis-related genes, including those involved in cell migration and invasion. Furthermore, epigenetic changes also impact the TME, including immune cells and stromal cells like CAFs, which can promote or suppress metastasis [188, 389, 390]. More recently, the booming epitranscriptomic field is uncovering the significance of RNA modifications and RNA modifiers in cancer progression and metastasis (Table 2). Furthermore, a recent finding describing that RNA modifications can influence CAFs to promote metastasis of neighborhood cells [391], supports the importance of epitranscriptomic alterations in tumor cell fate, not only by inner but also by outer influences from the TME. Altogether, these *epi-regulations* control the function of the genome, by modulating transcription activation, enhancers, transcript stability, translation rate, or molecular interactions. Post-translational modifications (e.g., acetylation, glycosylation, ubiquitination, SUMOylation, methylation) can also regulate EMT effectors affecting their stability and activity, offering also therapeutic opportunities [392–394]. Moreover, during the metastatic cascade, tumor cells interact with the immune system and must escape from recognition. Strikingly, although out of the scope of this review, recent studies have started to unveil how cancer cells communicate with immune cells to promote their metastatic potential [395, 396].

Over the past decade, giant leaps have been made in the field of epitranscriptomics. Improvement of detection technologies has allowed the characterization of the global landscape of several RNA modifications, as well as its dynamic nature across different cellular scenarios. However, several findings are still missing independent validation by different teams. Moreover, further efforts should be made to develop novel strategies for orthogonal validation of RNA modification profiles. Importantly, research approaches focused on identifying the molecular mechanisms upstream and downstream of dysregulated DNA and RNA modifiers, as well as comprehensive assessment of clinical implications, are needed to unravel novel clinical opportunities.

It is also essential to take advantage of recent single-cell technologies to disentangle intratumoral heterogeneity also from an epigenetic and epitranscriptomic perspective. Strong evidences support the relevance of epigenetic reprogramming as a driving force in the dynamic transcriptomic heterogeneity in cancer [397]. Using these strategies, we could zoom in on to the cancer epigenome and epitranscriptome and explore the *epi-players* that regulate different aspects of tumor heterogeneity and the intricate mechanisms governing metastasis or drug response at the highest resolution. Single-cell approaches could be useful, for example, to detect metastatic clones and identify therapeutic vulnerabilities that can be tackled to improve the clinical benefits of current therapies or to develop novel strategies. Recently developed methodologies for studying the epigenome at single-cell resolution include multiplexed single-cell reduced-representation bisulfite sequencing (scRRBS) to assess DNA methylation [398, 399], and the transposase-accessible chromatin sequencing assay at single-cell level (scATAC-seq) to measure DNA accessibility [400, 401]. Also, combining scATAC-seq with single-cell RNA sequencing (scRNA-seq), dynamic cell states modulated by epigenetic mechanisms could be defined.

**Fig. 4:**
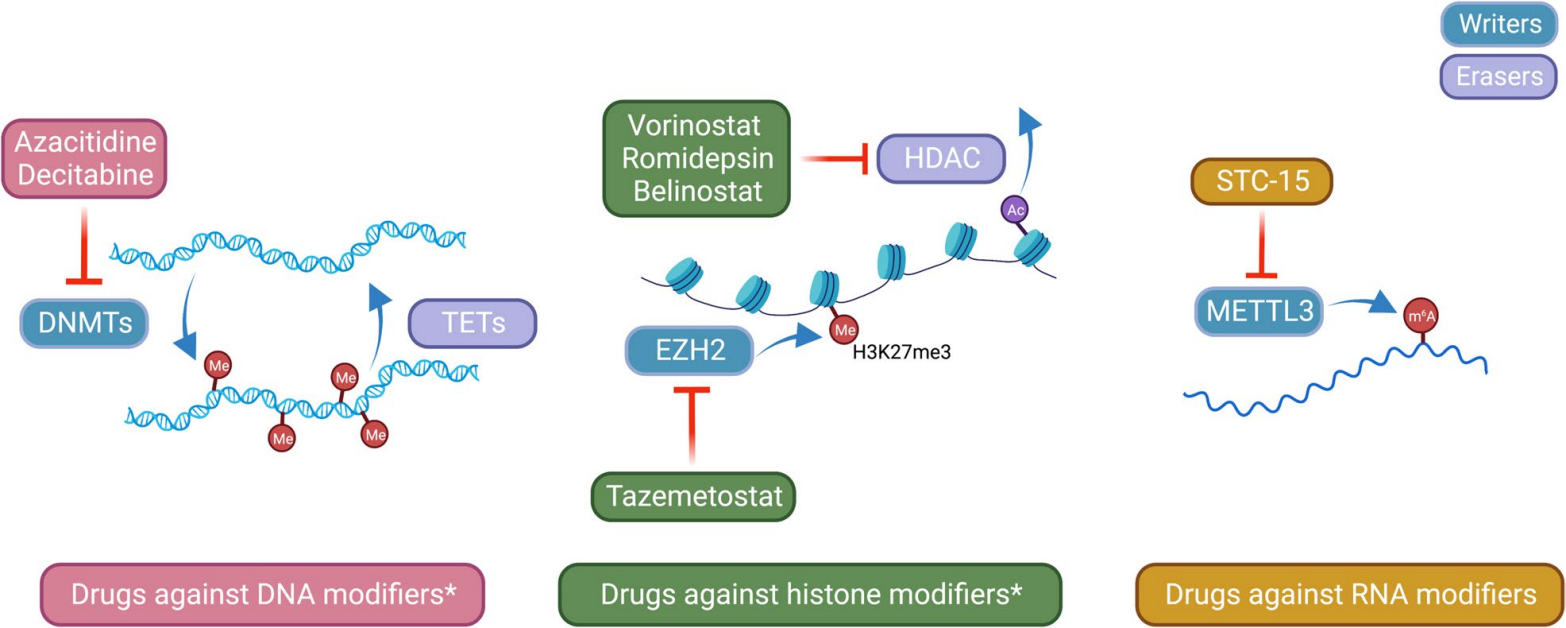
Key drugs against DNA, histone, and RNA modifiers. *Only drugs approved by the Food and Drug Administration (FDA) are depicted. For RNA modifiers, STC-15 is the first molecule specifically targeting an RNA methyltransferase enzyme to enter clinical trials (NCT05584111). Abbreviations: Ac, acetylation; DNMTs, DNA methyltransferases; EZH2, enhancer of Zeste 2 polycomb repressive complex 2 subunit; H3K27me3, histone H3 lysine 27 trimethylation; HDAC, histone deacetylase; m^6^A, N6-methyladenosine; Me, methylation; METTL3, N6-adenosine-methyltransferase 3; TETs, ten-eleven translocation 1/2/3. Created with BioRender.com

Finally, the fundamental role of epigenetic mechanisms in shaping genome function, coupled with the epigenetic dysregulation that occurs in cancer, has made the epigenetic machinery an attractive target for drug development, particularly given the plasticity of the epigenetic modifications. Thus, development of *epidrugs*, drugs that target enzymes involved in epigenetic regulation of genome function, as a strategy for tackling cancer is an active field of research. The rationale behind the use of epigenetic drugs lies in the possibility of restoring a balanced transcriptional landscape by modifying the chromatin states. Current epigenetic drugs target enzymes that introduce (writers), recognize (readers), and remove (erasers) epigenetic marks to DNA or core histones, a topic recently reviewed by our group [402]. Some of these drugs are already used in the clinical practice, such as the DNMT inhibitors (DNMTi) azacitidine and decitabine for the treatment of acute myeloid leukemia (AML) and myelodysplastic syndrome (MDS); and the HDAC inhibitors (HDACi) belinostat for peripheral T-cell lymphoma treatment (PTCL), or vorinostat and romidepsin to treat cutaneous T-cell lymphoma (CTCL) (Fig. 4). However, despite the significant benefits of DNMTi and HDACi in the clinical management of several hematological malignancies, lack of target selectivity and off-target effects of these inhibitors and unfavorable pharmacokinetic and pharmacodynamics have been detected. More recently, the power of *epidrugs* as modulators of sensitivity to other antineoplastic therapies is being explored, opening new opportunities for using them in solid tumors. In this regard, the ability of the epigenetic drugs to reverse many processes that tumors engage to evade immune-mediated destruction has promoted the development of clinical trials that combine immune checkpoint inhibitors with *epidrugs* to overcome some of the current limitations of immunotherapy and improve the clinical benefits [402]. Interestingly, in gastric cancer, it has been shown that the natural compound peperomin E exhibits DNMTi activities and drives hypomethylation of metastasis suppressor genes, leading to cell migration and metastasis inhibition [403].

In addition to these broad epigenetic reprogrammers such as DNMTi and HDACi, the identification of mutations in *EZH2*, *IDH1*, or *IDH2* in specific tumor types opened new opportunities for precision medicine and boosted the interest in the development of EZH2 and IDH inhibitors. Successful examples include the EZH2 inhibitor tazemetostat that has been approved for the treatment of follicular lymphoma and epithelioid sarcoma (Fig. 4), the IDH1 inhibitor ivosidenib indicated for the treatment of AML or cholangiocarcinoma patients harboring IDH1 mutations, and the IDH2 inhibitor enasidenib approved as therapeutic agent for IDH2-mutated AML patients [402]. As previously commented, inhibition of TET demethylating catalytic activity in IDH1/IDH2‐mutant cells disrupts the epigenome and promotes cancer by altering global DNA methylation; thus, synergistic inhibition of IDH and DNMT has been assessed in clinical trials to restore DNA methylation landscape, leading to the recent approval of the use of ivosidenib in combination with azacitidine in newly diagnosed IDH1-mutated AML patients [402]. Another example of the use of *epidrugs* in the context of precision oncology is the inhibitors of the histone demethylase LSD1. This is a promising therapy for Ewing sarcoma, since LSD1 is a critical functional partner of EWS/FLI, the driver fusion protein in this tumor type that arises from the characteristic t(11;22) translocation. The LSD1 inhibitor seclidemstat is currently under study in patients with Ewing sarcoma or Ewing-related sarcomas with FET-family translocations. Moreover, considering that several studies have demonstrated that the demethylase function of LSD1 is not restricted to histones, and DNMT1 is one of these non-histone targets, several clinical trials are testing the combination of LSD1 inhibitors and DNMTi for the treatment of hematological malignancies [402]. Importantly, synergy between epigenetic drugs and other anticancer therapies, as well as their potential to reverse acquired therapy resistance by modulating the sensitivity of cancer cells to other treatments (including not only immunotherapy, but also chemotherapy, radiation therapy, hormone therapy, and molecularly targeted therapy), are also giving rise to new possibilities for maximizing the efficacy of cancer treatments. For instance, cancer cells that have acquired resistance to targeted therapies or chemotherapy often exhibit specific epigenetic changes, and by targeting these alterations, epigenetic drugs may sensitize resistant cells to previously ineffective treatments. Additionally, considering that epigenetic alterations can cooperate with genetic mutations or signaling pathway dysregulation in driving cancer progression, combining epigenetic drugs with targeted therapies that address these genetic abnormalities can have synergistic effects and improve patient outcomes. Remarkably, the contribution of epigenetics to precision oncology is not only limited to the *epidrug* field. Epigenetic characterization of human tumors has revealed a plethora of cancer-specific epigenetic marks or signatures of potential use as biomarkers along the course of the disease. Several DNA methylation-based assays of clinical utility are already commercially available to aid precise diagnosis, monitor disease recurrence, or predict therapy response to further support clinical decision-making and ultimately improve patient clinical management and outcomes [402].

The epitranscriptomic field has started to be also explored with therapeutic purposes. Dysregulation in cancer and reversibility of the epitranscriptomic marks are also the two features that have drawn the attention of pharmaceutical companies. The most advanced example is the development of METTL3 inhibitors, with multiple compounds disclosed by Accent Therapeutics and STORM Therapeutics. In November 2022, the oral METTL3 inhibitor STC-15 (STORM Therapeutics) was the first molecule specifically targeting an RNA methyltransferase enzyme to enter clinical development (Fig. 4). The phase I study (NCT05584111) will assess safety and tolerability, pharmacokinetics, pharmacodynamics, and anti-tumor efficacy of STC-15 in adult patients with advanced solid tumors.

Although advances in the field are tremendous, there are still many challenges to overcome in the development of effective epi-based therapies for cancer (and metastasis). One of the major obstacles is the above commented intratumoral heterogeneity, as different cell populations could have different metastatic potential and/or sensitivity to treatment; and then pre-existing non-target populations could persist after treatment and result in treatment resistance or disease relapse. Thus, considering on the one hand the effects of epi-based therapies in reshaping the landscape of DNA and RNA modifications, and on the other the critical role of tumor heterogeneity in drug response, the multiomic analysis of malignant cells and the TME at single-cell resolution will be key to deciphering and understanding the biological effects of drugs targeting epigenetic and epitranscriptomic enzymes, and consequently to improving therapeutic strategies. Most of the epi-modifications/epi-modifiers herein described are potential clinical biomarkers for diagnosis or prognosis, but understanding better the role of RNA and DNA modifications in cancer metastasis will be crucial for developing effective therapies to prevent or treat metastatic disease.
